# Associations of the Muscle Strength Index with Overweight/Obesity, Elevated Blood Pressure, and Their Comorbidity in Chinese Children and Adolescents During Two Decades

**DOI:** 10.3390/jcm15072712

**Published:** 2026-04-03

**Authors:** Ruolan Yang, Shan Cai, Jiajia Dang, Tianyu Huang, Jiaxin Li, Yunfei Liu, Kaiheng Zhu, Ziyue Sun, Yang Yang, Jun Ma, Yi Song

**Affiliations:** Institute of Child and Adolescent Health, School of Public Health, Peking University, Beijing 100191, China; 2010306116@stu.pku.edu.cn (R.Y.); caishan@pku.edu.cn (S.C.); dangjj@bjmu.edu.cn (J.D.); tyhuang@pku.edu.cn (T.H.); lijiaxin@bjmu.edu.cn (J.L.); melatonin@pku.edu.cn (Y.L.); 2516397050@bjmu.edu.cn (K.Z.); sunziyue@bjmu.edu.cn (Z.S.); 2516395133@bjmu.edu.cn (Y.Y.); majunt@bjmu.edu.cn (J.M.)

**Keywords:** overweight/obesity, elevated blood pressure, muscle strength index, child

## Abstract

**Background**: The rising prevalence of childhood overweight/obesity (OWOB) and elevated blood pressure (EBP) parallels a global decline in muscular fitness. However, evidence linking whole-body muscular strength to the comorbidity of these cardiometabolic risks remains scarce. **Methods**: Data were obtained from five nationally representative waves of the Chinese National Survey on Students’ Constitution and Health (CNSSCH, 2000–2019), including 1,072,404 children and adolescents aged 7–18 years. A novel Muscle Strength Index (MSI) was developed by integrating handgrip strength (HGS) and standing broad jump (SBJ), standardized for body weight and height, respectively. Generalized linear mixed-effects models (GLMMs) with restricted cubic splines (RCS) were first applied to characterize dose–response associations. Subsequently, categorical analyses and forest plots were conducted to quantify risks of OWOB, EBP, and their comorbidity across five waves and subgroups. Sex-specific normative reference curves were established using the LMS method, and population-attributable fractions (PAFs) were estimated to assess the potential public health benefits of improving muscular strength. **Results**: Between 2000 and 2019, the prevalence of OWOB, EBP, and comorbidity increased markedly, reaching 25.80%, 12.23%, and 4.83% in 2019, and are projected to rise further to 37.88%, 20.16%, and 10.01% by 2030. Over the same period, mean MSI increased from 2000, peaked in 2005, and subsequently declined by 2019 with the values for boys and girls, being 1.73, 1.75, 1.63 and 1.46, 1.49, 1.41, respectively. Dose–response analyses revealed consistent L-shaped associations, with the greatest risk reductions observed when moving from low to moderate MSI levels. In 2019, participants with low MSI had higher odds of OWOB (OR 4.81, 95% CI 4.65–4.97), EBP (OR 1.42, 95% CI 1.36–1.49), and comorbidity (OR 3.49 95% CI 3.26–3.73) compared with those at middle levels. PAF analyses indicated that improving MSI to at least the 40th percentile could potentially avert 43.5% of OWOB cases, 12.3% of EBP cases, and 48.2% of comorbidity cases. The highest potential benefits were observed in northern and northeastern provinces, particularly Tianjin and Heilongjiang. **Conclusions**: Chinese children and adolescents face a dual burden of rising cardiometabolic comorbidity and declining muscular strength. Muscular strength demonstrates a strong nonlinear protective association with OWOB, EBP, and their co-occurrence. Targeted improvement among those with low muscular strength may substantially reduce future cardiometabolic burden.

## 1. Introduction

Childhood and adolescence represent a critical developmental window for shaping lifelong health phenotypes and establishing enduring lifestyle habits [1]. According to the American Heart Association’s Life’s Essential 8 framework, body weight and blood pressure are two fundamental, non-invasive, and easily monitored indicators of cardiovascular health [2,3]. However, the global prevalence of overweight/obesity (OWOB) and elevated blood pressure (EBP) in youth has escalated alarmingly in recent decades. Evidence indicates a 1.5-fold increase in the global prevalence of childhood obesity from the 2000–2011 period to 2012–2023 [4], alongside a nearly 80% rise in the relative rate of childhood EBP between 2000 and 2015 [5]. Crucially, OWOB and EBP often co-occur as cardiometabolic comorbidities, largely due to shared biological mechanisms such as insulin-mediated sympathetic nervous system activation [6,7,8]. The co-occurrence of OWOB and EBP in childhood elevates the risk not only for early cardiometabolic abnormalities but also for subsequent cardiovascular disease, diabetes, and related complications in adulthood, making early identification and intervention a public health imperative [9]. Despite the urgency, systematic evidence regarding the long-term trends and co-occurrence of OWOB and EBP among Chinese children and adolescents remains limited.

Concurrently, muscular strength is increasingly recognized as a powerful marker of current and future health, inversely associated with adiposity, cardiovascular risk, and all-cause mortality [10,11]. Handgrip strength (HGS) and the standing broad jump (SBJ) are widely used measures in children and adolescents, serving as simple and reliable indicators of upper- and lower-limb muscular fitness, respectively [12,13]. However, a recent study based on the China National Survey on Students’ Constitution and Health (CNSSCH), which included approximately 1.33 million students aged 7–22 years from 2000 to 2019, revealed a marked decline in HGS and SBJ performance after 2010, which was inversely correlated with concurrent increases in body weight [14]. Against the background of rapidly rising global prevalence of OWOB and EBP in youth, this downward trend in muscular strength warrants urgent attention. This downward trend, occurring against the backdrop of an obesity epidemic, exacerbates the “dual burden” of poor health in youth.

While the protective associations of muscular strength against individual cardiovascular risk factors are well-documented [15,16], crucial knowledge gaps remain. First, prior research has largely depended on single indicators, such as HGS or SBJ. Yet, without standardization, these metrics are inherently confounded by anthropometric variables, which may obscure true functional capacity, especially in youth with extreme body sizes. Consequently, size-standardized indices integrating both upper- and lower-body strength are indispensable for an accurate and rigorous evaluation of comprehensive muscular health. Second, the association between muscular strength and the specific comorbidity of OWOB and EBP is largely unexplored. In particular, the shape of this association—specifically whether a nonlinear dose–response relationship exists—remains under-investigated. Additionally, most existing research has concentrated on adults or elderly populations [11], and pediatric studies are often limited by small sample sizes, lack of national representativeness.

To address these gaps, this study leveraged data from five waves of the CNSSCH (2000–2019), a large-scale, nationally representative survey. We developed a novel composite Muscular Strength Index (MSI), integrating body-size-standardized HGS and SBJ, to provide a holistic assessment of muscular fitness. The specific objectives of this study were to: (1) delineate the secular trends in OWOB, EBP, their comorbidity, and muscular strength over two decades; (2) quantify the dose–response associations between MSI and these cardiometabolic outcomes; and (3) estimate the population-attributable fractions (PAFs) to evaluate the potential public health benefits of improving muscular strength among Chinese youth.

## 2. Materials and Methods

### 2.1. Study Design and Data Source

Data were obtained from the Chinese National Survey on Students’ Constitution and Health (CNSSCH), a nationally representative, school-based repeated cross-sectional survey conducted every five years across 31 provinces in mainland China [17]. Five survey waves (2000, 2005, 2010, 2014, and 2019) were included. The CNSSCH uses a multistage, stratified cluster sampling design. In each survey cycle, provinces serve as primary sampling units. Within each province, schools are stratified by urban–rural residence and regional socioeconomic level. Classes are randomly selected within sampled schools, and all students in selected classes are invited to participate. Measurement protocols and instruments were consistent across survey waves [18]. The study protocol was approved by the Biomedical Ethics Committee of Peking University Health Science Center (IRB00001052–19095). All participants and their guardians provided informed consent in accordance with national regulations.

### 2.2. Study Population

Data from 2000–2019 were pooled. Participants were eligible if they were aged 7–18 years and had complete data on demographic variables, anthropometric measurements, blood pressure, handgrip strength, and standing broad jump. Individuals with biologically implausible values were excluded according to established screening criteria. Analyses were restricted to participants of Han ethnicity with valid urban–rural classification codes. After exclusions, 1,072,404 participants were included (Figure S1).

### 2.3. Measurements and Definitions

#### 2.3.1. Anthropometric and Blood Pressure Assessments

Height and weight were measured by trained health professionals following standardized protocols. Height was recorded to the nearest 0.1 cm with participants standing barefoot and wearing light clothing. Weight was measured to the nearest 0.1 kg using standardized electronic or lever scales, which were calibrated daily with 100 g reference weights for sensitivity and 10, 20, and 30 kg weights for accuracy. Three repeated measurements were taken for each parameter, and the mean values were used for analysis.

Blood pressure (BP) was measured using a mercury sphygmomanometer after participants had rested in a seated position for at least 10 min. Systolic BP (SBP) was determined by the onset of the first Korotkoff sound, and diastolic BP (DBP) by the disappearance of the fifth sound. Three consecutive readings of SBP and DBP were obtained during a single visit, and the average of the three measurements was used in the analysis.

#### 2.3.2. Muscular Strength Assessments

Upper-body muscular strength was evaluated using Handgrip strength (HGS), measured with an electronic dynamometer (WCS-100, Beijing Xindonghuateng Sports Facility Co., Ltd., Beijing, China). Participants adjusted the grip span to ensure a comfortable hand fit and performed two maximal-effort trials, with results recorded to the nearest 0.1 kg. The higher of the two readings was used for analysis. Lower-body muscular strength was assessed by standing broad jump (SBJ), conducted on level sand pits or soft ground under the supervision of trained physical education teachers. Each participant completed three attempts, and the longest distance, measured to the nearest centimeter, was recorded for analysis.

#### 2.3.3. Overweight/Obesity (OWOB)

Body mass index (BMI) was calculated as body weight (kg) divided by height squared (kg/m^2^). OWOB were assessed using BMI according to the national health standard for screening school-age children and adolescents (WS/T 586-2018) [19]

#### 2.3.4. Elevated Blood Pressure (EBP)

EBP was defined according to the national screening reference (WS/T 610-2018) for children and adolescents aged 7–18 years [20].

#### 2.3.5. Comorbidity

Comorbidity was defined as co-occurrence of OWOB and EBP.

#### 2.3.6. Muscular Strength Index (MSI)

To capture overall muscular fitness, we constructed the Muscular Strength Index (MSI) by integrating upper- and lower-body strength. Because absolute strength in children and adolescents is strongly influenced by body size, handgrip strength (HGS) and standing broad jump (SBJ) were standardized to body weight and height, respectively, to reduce anthropometric confounding [21,22]. The relative strength indices were calculated as follows:

$$Relative\;grip\;strength=\frac{Handgrip\;strength\;(\mathrm{kg})}{Weight\;(\mathrm{kg})}$$

$$Relative\;standing\;broad\;jump=\frac{Standing\;broad\;jump\;(m)}{Height\;(m)}$$

The MSI was defined as the sum of relative strength indices [23]:

MSI=Relative grip strength+Relative standing broad jump

An unweighted sum was used to give equal contribution to upper- and lower-body strength. This dimensionless composite measure reduces body-size confounding while remaining simple and suitable for large-scale epidemiological studies.

### 2.4. Statistical Analysis

#### 2.4.1. Descriptive Analysis

Continuous variables were summarized as means with standard errors (SEs), and categorical variables as frequencies and percentages. Normality of continuous variables was assessed using Q–Q plots (Figure S2). Temporal trends in the prevalence of OWOB, EBP, and comorbidity were evaluated using the Cochran–Armitage trend test, while trends in continuous measures (HGS, SBJ, and MSI) were assessed using the Jonckheere–Terpstra test.

#### 2.4.2. Trend Analysis and Future Trend Projections

Temporal trends (2000–2019) and future prevalence up to 2030 were analyzed using generalized linear models (GLMs) with a binomial distribution and logit link. Survey year was modeled using natural cubic splines with 3 degrees of freedom to account for potential non-linearity. The fitted models were then used to project annual prevalence for 2020–2030. To account for sampling variability, 95% confidence intervals (CIs) were estimated using bootstrap resampling with 100 iterations.

#### 2.4.3. Generalized Linear Mixed-Effects Models (GLMMs)

Given the multistage clustered sampling design of the CNSSCH, we first estimated intraclass correlation coefficients (ICCs) using null mixed-effects models. Since ICCs for EBP and comorbidity exceeded 0.1 (Table S1), indicating substantial school-level clustering, all subsequent association analyses were conducted using GLMMs with logit links and random intercepts for schools.

#### 2.4.4. Association Analysis

We first examined the associations between MSI (continuous) and health outcomes. To capture potential nonlinearity, natural cubic splines with three knots (placed at the 10th, 50th, and 90th percentiles) were incorporated into the GLMMs, with odds ratios (ORs) calculated relative to the median MSI. Models were adjusted for age group, sex, residence, and survey year. In subgroup analyses, the stratification variable was excluded from the adjustment set.

For categorical analyses, MSI was classified into five levels using sex- and age-specific percentiles derived from the pooled 2000–2019 dataset to ensure comparability across waves: Low (<20th), Mid-Low (20th–40th), Mid (40th–60th, reference), Mid-High (60th–80th), and High (≥80th). Wave-stratified GLMMs were fitted to evaluate the temporal stability of these associations.

#### 2.4.5. Population Attributable Fraction (PAF)

To estimate the potential public health benefits under the current population structure, we established a contemporary reference standard using the 2019 survey data. Age- and sex-specific MSI percentiles were derived using the LMS (Lambda–Mu–Sigma) method, which models skewness, median, and coefficient of variation via Box–Cox transformation.

The 40th percentile was selected as the counterfactual threshold, as our categorical analyses consistently identified the Low (<20th) and Mid-Low (20th–40th) groups as risk categories associated with elevated odds of adverse outcomes, whereas the Mid-High and High groups demonstrated protective effects compared with the reference group. For PAF estimation, age- and sex-specific cutoffs corresponding to the 40th percentile were derived from the 2019 LMS reference distribution to reflect the current population structure. Consequently, PAFs were calculated under a hypothetical scenario in which individuals in the risk groups (Low and Mid-Low, <40th percentile) were shifted to at least the 40th percentile according to the 2019 reference values. Estimates were derived from adjusted GLMMs comparing predicted probabilities under observed versus counterfactual exposure distributions. Robust 95% confidence intervals were generated using cluster-level bootstrap resampling (100 iterations at the school level). Provincial PAFs were also estimated to assess geographic heterogeneity. These estimates should be interpreted as hypothetical and do not imply causal relationships.

All analyses were performed using R software (version 4.3.1). Statistical significance was defined as a two-sided *p* < 0.05.

### 2.5. Sensitivity Analysis

To test the robustness of our findings, two sets of sensitivity analyses were conducted. First, OWOB was alternatively defined according to the CDC 2000 growth charts, with overweight defined as BMI ≥85th to <95th percentile and obesity as ≥95th percentile [24,25]. Second, we repeated both the restricted cubic spline analyses and the categorical GLMMs using individual metrics of relative handgrip strength and relative standing broad jump.

## 3. Results

### 3.1. Characteristics of the Study Population

As shown in Table 1, a total of 1,068,408 participants aged 7–18 years were included across five survey waves (2000–2019). The distributions of sex and residence remained stable over time. From 2000 to 2019, height, weight, and BMI increased significantly (all *p* < 0.001). During the same period, the prevalence of OWOB rose from 10.0% to 25.8%, while EBP and comorbidity increased from 9.1% to 12.2% and 1.7% to 4.8%, respectively (all *p* < 0.001). Conversely, SBJ and MSI declined significantly across the study period (all *p* < 0.001), indicating a divergence between increasing cardiometabolic profiles and declining muscular strength.

### 3.2. Long-Term Trends and Projections of OWOB, EBP, and Comorbidity

**Figure 1:**
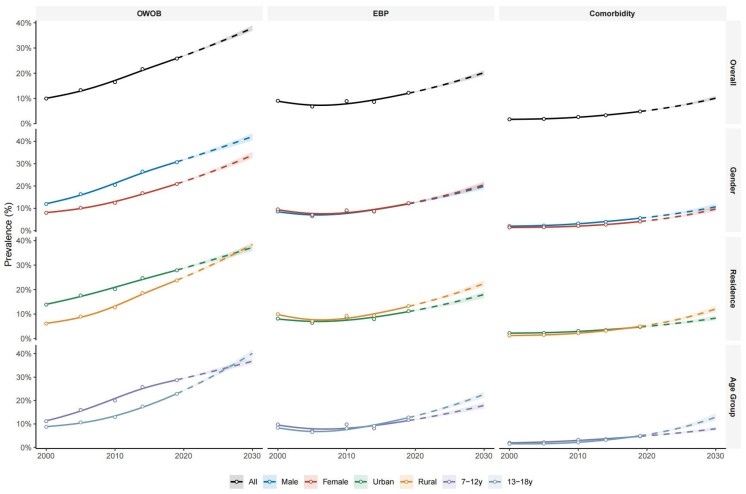
Temporal trends and future projections of OWOB, EBP, and comorbidity prevalence among Chinese children and adolescents aged 7–18 years (2000–2030). The circles represent the crude observed prevalence rates derived from the five national surveys (2000–2019). The lines indicate the modeled trajectories estimated using logistic regression models incorporating natural cubic splines (df = 3). Solid lines denote the fitted trends for the historical period (2000–2019), while dashed lines represent the projected trends for the future period (2020–2030). Shaded ribbons represent 95% confidence intervals derived from bootstrap resampling (100 iterations). Results are stratified by sex, residence, and age group. OWOB: overweight/obesity; EBP: elevated blood pressure.

Figure 1 shows that between 2000 and 2019, the prevalence of OWOB, EBP and comorbidity increased markedly among Chinese children and adolescents. By 2019, the prevalence of OWOB, EBP, and their comorbidity had reached 25.81% (95%CI: 25.62–26.00), 12.25% (95%CI: 12.11–12.39), and 4.84% (95%CI: 4.74–4.93), respectively. Projections indicate that this upward trend is likely to accelerate. Model-based projections suggested continued increases by 2030, with OWOB estimated to reach 37.91% (36.83–38.97), EBP 20.22% (19.15–21.21), and comorbidity 10.11% (9.46–11.03) (Table S2). Trends were consistent across sex, age group, and residence subgroups, and the findings were in line with our sensitivity analyses (Figure S3, Table S3).

### 3.3. Temporal Patterns of Muscular Strength

**Figure 2:**
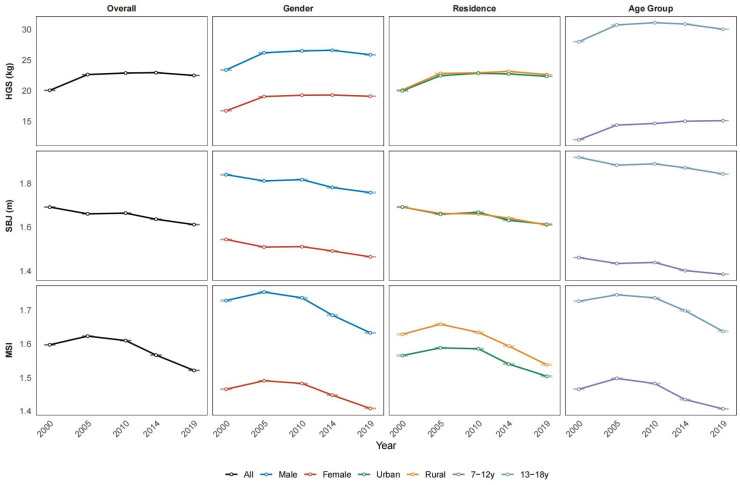
Temporal trends in handgrip strength, standing broad jump, and muscle strength index among Chinese children and adolescents aged 7–18 years, 2000–2019. Data are presented as means with 95% confidence intervals (CIs). The points represent the mean values for each survey year, and the vertical error bars indicate the 95% CIs. Trends are stratified by sex (male vs. female), residence (urban vs. rural), and age group (7–12 years vs. 13–18 years). HGS: handgrip strength; SBJ: standing broad jump; MSI: muscle strength index.

Figure 2 shows that HGS increased modestly between 2000 and 2014 before declining by 2019, whereas SBJ declined steadily across the study period. As a composite measure, MSI showed a slight increase in the early waves followed by a sustained decline through 2019. Boys consistently demonstrated higher HGS, SBJ, and MSI than girls, and urban adolescents and older age groups had lower MSI compared with their rural and younger counterparts. Specifically, mean HGS in boys increased from 23.40 kg (95%CI: 23.32–23.48) in 2000 to a peak of 26.60 kg (95%CI: 26.52–26.68) in 2014, then declined to 25.88 kg (95%CI: 25.80–25.95) in 2019. A similar pattern was observed in girls, whose HGS peaked in 2014 at 19.30 kg (95%CI: 19.26–19.34), and then decreased to 19.10 kg (95%CI: 19.06–19.14) in 2019. For SBJ, boys’ performance was highest in 2000 at 1.84 m (95%CI: 1.84–1.84), then declined to 1.76 m (95%CI: 1.76–1.76) in 2019, whereas girls declined steadily from 1.54 m (95%CI: 1.54–1.55) in 2000 to 1.46 m (95%CI: 1.46–1.47) in 2019. The MSI rose from its 2000 values (boys: 1.73 [1.73–1.73]; girls: 1.47 [1.46–1.47]), reached its peak in 2005 (boys: 1.75 [1.75–1.76]; girls: 1.49 [1.49–1.49]), and eventually fell to 1.63 (1.63–1.64) for boys and 1.41 (1.41–1.41) for girls in 2019 (Table S3). Overall, population-level muscular strength weakened over time, particularly in the most recent wave.

### 3.4. Associations Between MSI and OWOB, EBP and Comorbidity

**Figure 3:**
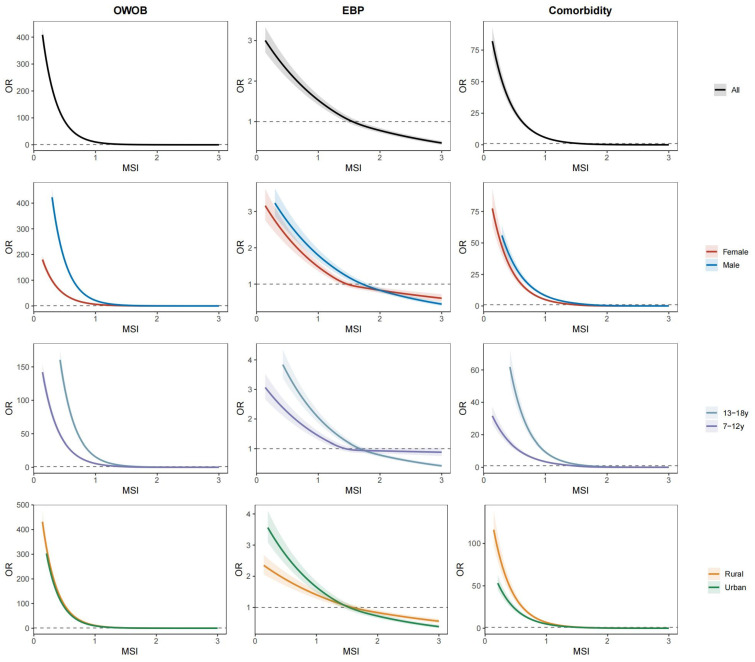
Dose–response associations of Muscle Strength Index (MSI) with the risks of OWOB, EBP, and their comorbidity among Chinese children and adolescents. Associations were estimated using generalized linear mixed-effects models (GLMMs) with restricted cubic splines. The solid lines represent the estimated odds ratios (ORs), and the shaded ribbons indicate the 95% confidence intervals. The models were adjusted for age group, sex, and residence, with school included as a random effect. The reference value for MSI was set at the median of the distribution. Knots were placed at the 10th, 50th, and 90th percentiles of the MSI distribution. The dashed horizontal line represents an OR of 1.0. OWOB: overweight/obesity; EBP: elevated blood pressure.

Figure 3 demonstrates the L-shaped curves for the dose–response relationships of MSI with the prevalence of OWOB, EBP, and comorbidity. Risk declined steeply at lower MSI levels and plateaued at higher levels of fitness. These associations were consistent across sex, age group and residence subgroups. Results of sensitivity analyses were similar to those described above (Figures S4 and S5).

**Figure 4:**
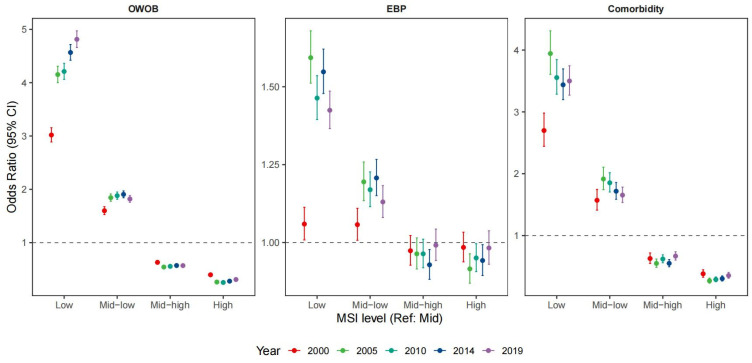
Temporal consistency of the associations between MSI levels and cardiometabolic risks across five survey waves (2000–2019). Data are presented as odds ratios (ORs) with 95% confidence intervals (CIs). Separate GLMMs were fitted for each survey year to assess the stability of associations over time. MSI was categorized into five levels based on sex- and age-specific percentiles: Low (<20th), Mid-low (20th–40th), Mid (40th–60th, Reference group), Mid-high (60th–80th), and High (≥80th). The dashed vertical line indicates an OR of 1.0. OWOB: overweight/obesity; EBP: elevated blood pressure.

Figure 4 shows that relative to the Mid group (40th–60th percentile), odds increased progressively across the Mid-Low and Low categories and decreased progressively across the Mid-High and High categories. This graded pattern aligns with the L-shaped nonlinear association identified in the spline analyses. These associations were stable across survey waves. In 2019, adjusted odds ratios for participants with Low MSI were 4.81 (4.66–4.97) for OWOB, 1.42 (1.37–1.49) for EBP, and 3.50 (3.27–3.75) for comorbidity, compared with the Mid group (Table S5). Results were consistent when OWOB was defined using CDC criteria (Figure S6, Table S6). Furthermore, sensitivity analyses using the individual muscular strength indicators, including relative handgrip strength (HGS/Wt) and relative standing broad jump (SBJ/Ht), showed similar graded inverse associations with all three cardiometabolic outcomes (Figures S7 and S8, Table S7).

### 3.5. Population-Level Impact of Improving Muscular Strength

**Figure 5:**
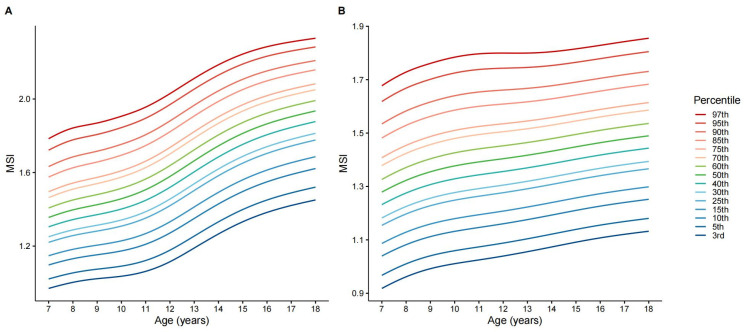
Sex-specific normative reference percentile curves for the Muscle Strength Index (MSI) among Chinese children and adolescents aged 7–18 years (based on 2019 CNSSCH data). The percentile curves were constructed using the Lambda–Mu–Sigma (LMS) method with a Box–Cox t-distribution (BCT) family in the gamlss package. The curves display the 3rd, 5th, 10th, 15th, 25th, 30th, 40th, 50th, 60th, 70th, 75th, 85th, 90th, 95th, and 97th percentiles for boys (**A**) and girls (**B**). Age was modeled using a logarithmic transformation (log(age)) to ensure smooth fit.

Sex-specific MSI percentile curves derived from the 2019 survey illustrated distinct developmental trajectories (Figure 5). MSI increased with age in both sexes during childhood, with a steeper rise among boys during adolescence, resulting in widening sex differences. These LMS-derived percentiles provide contemporary normative reference values for muscular strength assessment (Tables S8 and S9).

**Figure 6:**
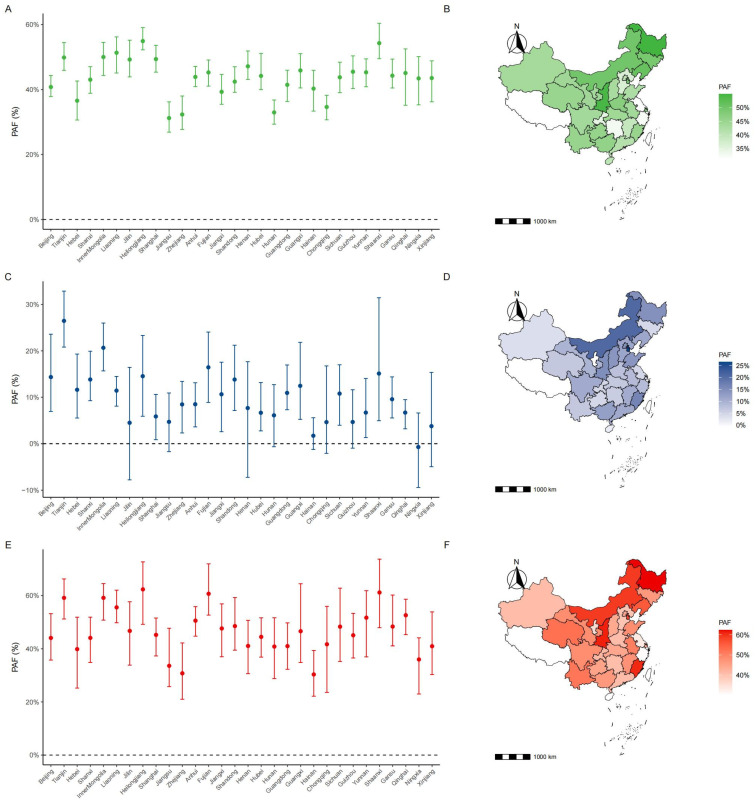
Population attributable fractions (PAFs) of cardiometabolic risks attributable to low muscular strength among Chinese children and adolescents by province (2019). The PAFs estimate the theoretical percentage of cases (OWOB, EBP, and Comorbidity) that could be prevented if children with low muscular strength (MSI <40th percentile) improved their strength to at least the moderate level (≥40th percentile). (**A**,**C**,**E**) Bar charts showing the estimated PAFs (dots) and 95% confidence intervals (vertical lines) for each province, ranked by geographic region. (**B**,**D**,**F**) Choropleth maps illustrating the geographic distribution of PAFs across mainland China. Panels (**A**) and (**B**) represent OWOB (green); Panels (**C**) and (**D**) represent EBP (blue); Panels (**E**) and (**F**) represent the Comorbidity of OWOB and EBP (red). OWOB: overweight/obesity; EBP: elevated blood pressure.

Figure 6 displays that when improving the MSI levels from low and middle-low (<40th percentile) to middle or above, students aged 7–18 years old in China exhibited significant reductions in OWOB, EBP and comorbidity of 43.5% (42.9–44.1%), 12.3% (11.2–13.2%) and 48.1% (46.6–49.6%), respectively. Provincial variation was observed (Figure 6). Northern and northeastern provinces exhibited the largest attributable fractions, including Tianjin (OWOB: 49.9% (45.9–54.5%); EBP: 26.5% (20.8–32.9%); comorbidity: 59.1% (51.2–66.2%)), and Heilongjiang (OWOB: 54.9% (52.2–59.1%); EBP: 14.5% (5.9–23.3%); comorbidity: 62.3% (49.2–72.6%)]) (Table S10). Results of sensitivity analyses were similar to those described above (Table S11).

## 4. Discussion

This study is the first to utilize a nationally representative dataset spanning two decades to systematically evaluate the “dual burden” of rising cardiometabolic risk and declining muscular strength among Chinese children and adolescents. To address the limitations of single-metric assessments, we developed a novel Muscular Strength Index (MSI), integrating upper- and lower-body performance standardized for body size. Using this index, we identified a robust L-shaped dose–response relationship, indicating that the most significant risk reductions occur when moving from low to moderate MSI levels. Crucially, our Population Attributable Fraction (PAF) analysis suggests that increasing MSI to at least the 40th percentile could theoretically prevent 43.5% of OWOB cases, 12.3% of EBP cases, and 48.1% of comorbidity cases, highlighting muscular strength as a high-potential target for preventive intervention.

Our findings confirm the escalating burden of obesity and EBP comorbidity, consistent with previous reports [26]. Concurrently, the trajectory of muscular strength presents a concerning decline. Unlike historical trends in the United States, where muscular strength showed a slight increase during the 20th century [27], the decline observed in our study mirrors recent epidemiological evidence from China, Lithuania, and the UK [14,28,29]. This international decline in pediatric muscular fitness likely reflects that the majority of adolescents fail to meet recommended physical activity levels [30]. The simultaneous rise in cardiometabolic risk factors and decline in protective muscular strength suggests a compounding public health challenge.

A key methodological contribution of this study is the development of the Muscular Strength Index (MSI), integrating upper- and lower-body performance into a unified framework. Handgrip strength (HGS) is widely used as a proxy for overall muscular fitness in pediatric and epidemiological research [31]; however, it primarily reflects upper-limb function and is strongly influenced by body mass, which may confound interpretation when comparing individuals of different sizes [32]. To provide a more comprehensive assessment of muscular fitness, we incorporated the standing broad jump (SBJ) as an indicator of lower-limb explosive power, a validated field-based test in children and adolescents [33]. Importantly, absolute SBJ performance is affected by anthropometric characteristics, including height. Prior studies using allometric modelling have demonstrated that body size significantly influences SBJ performance and that scaling for anthropometric variables improves comparability across individuals of different statures and growth stages [34]. In line with this evidence, we standardized HGS by body weight and SBJ by height to partially account for size-related variation and enhance cross-individual comparability. By synthesizing these standardized metrics, the resulting MSI provides a composite and dimensionless indicator of whole-body muscular performance, designed to reduce body-size confounding while retaining practical applicability in large-scale field settings.

Using this MSI metric, we observed a robust inverse association with OWOB, EBP and comorbidity across all subgroups. These associations remained stable over the five survey waves, highlighting their generalizability. Our results also align closely with findings from diverse pediatric populations worldwide. For example, cross-sectional data from European children link lower muscular fitness to higher cardiometabolic risk [35]. Similarly, Australian longitudinal cohorts show that early-life muscle strength independently predicts lower blood pressure and adiposity during adolescence [36]. Evidence from US cohorts further confirms that body-mass-normalized strength is strongly associated with reduced cardiometabolic risk clustering [22]. By analyzing a two-decade national dataset, our study reinforces this international consensus, demonstrating that the protective role of muscular strength transcends ethnic and geographic boundaries. Several biological mechanisms may underlie this protective association. Skeletal muscle is a metabolically active tissue that plays a central role in glucose uptake and insulin sensitivity and modulates vascular and inflammatory pathways through the secretion of myokines [37,38,39]. In addition, greater muscular strength often reflects higher engagement in moderate-to-vigorous physical activity, which improves vascular function and autonomic balance by reducing sympathetic tone and enhancing parasympathetic regulation [40,41,42].

Beyond establishing an inverse association, our spline analyses further demonstrated an L-shaped dose–response relationship between MSI and all outcomes. Consistent with findings in older Chinese adults [43], this nonlinear pattern shows that cardiometabolic risk declines steeply from low to moderate MSI levels and then plateaus at higher levels. The categorical analyses corroborated this graded pattern. Together, these findings suggest the presence of a functional threshold, whereby the greatest marginal health gains may be achieved by improving muscular strength among children and adolescents with initially low MSI.

To translate these associations into public health practice, we further established the first contemporary sex-specific normative reference curves for MSI in Chinese youth based on the 2019 data. Boys demonstrated a marked acceleration in strength development during puberty, whereas girls exhibited a more linear progression, underscoring puberty as a critical biological window and supporting the need for sex-specific intervention strategies. These reference percentiles provide a practical tool for early identification of children with low muscular fitness.

Our projection analysis depicts a concerning future scenario that without effective intervention, the prevalence of OWOB, EBP, and their comorbidity is projected to reach 37.91%, 20.22%, and 10.11%, respectively, by 2030. Using the 40th percentile cutoff derived from normative references, our PAF analysis further suggests that 48.1% of comorbidity cases may be attributable to low muscular strength. Although PAF estimates assume a causal interpretation and should be viewed cautiously, these findings indicate that improving muscular strength could yield substantial population-level benefits.

To evaluate the practical feasibility of improving muscular strength, evidence from intervention studies is informative. Compared with general physical activity or lifestyle-based weight management programmes, which have shown variable or modest effects on body composition and cardiometabolic outcomes in youth, structured strength-training interventions have consistently demonstrated improvements in muscular fitness. Meta-analyses indicate that school-based strength-training programmes can significantly enhance muscular strength and power within several weeks of supervised training. While long-term cardiometabolic benefits warrant further study, incorporating muscle-strengthening activities into school curricula appears feasible and scalable. Geographically, the potential benefits were unevenly distributed, with the highest PAFs observed in northern and northeastern provinces including Tianjin and Heilongjiang, highlighting the importance of geographically targeted interventions.

Translating these findings into practice requires coordinated policy action. First, national health surveillance systems should integrate the MSI into routine school health examinations to enable longitudinal monitoring of musculoskeletal health. Second, school physical education curricula should be revised to include structured muscle-strengthening activities [44], as fewer than 40% of students currently meet WHO recommendations for such activities [45]. Incorporating resistance training (e.g., bodyweight exercises, elastic bands) alongside aerobic activity could maximize cardiometabolic benefits [46].

This study has several strengths, including its large, nationally representative sample size spanning two decades, the development of a novel, body-size-standardized MSI, and the use of the LMS method to generate robust reference values. However, limitations must be acknowledged. First, the repeated cross-sectional design precludes causal inference, as the data represent independent samples rather than tracking the same children over time. However, the consistency across five survey waves strengthens the evidence for an association. Second, blood pressure was measured at a single visit, potentially overestimating EBP prevalence due to the “white-coat effect” [47]. Third, the MSI was constructed using only HGS and SBJ; incorporating measures of core strength would further refine the index. Fourth, PAF estimates were based on odds ratios rather than risk ratios and should be interpreted with caution until validated in prospective studies [48]. Finally, while we adjusted for key demographic confounders, residual confounding from unmeasured factors such as dietary intake and genetic predisposition cannot be ruled out.

## 5. Conclusions

In summary, the early 21st century has witnessed a rapid increase in cardiometabolic risk, coinciding with a marked decline in muscular strength among Chinese children and adolescents. Muscular strength exhibits a strong, L-shaped protective association with OWOB, EBP, and their comorbidity. Our findings imply that improving muscular strength in the bottom 40% could theoretically avert a significant fraction of the impending cardiometabolic disease burden. These findings suggest that early identification and targeted improvement of low muscular strength may represent a strategic and potentially high-impact approach to mitigating future cardiometabolic burden in youth.

## Figures and Tables

**Table 1:** Demographic characteristics, anthropometric profiles, and cardiometabolic risk prevalence among Chinese children and adolescents aged 7–18 years, 2000–2019.

	2000	2005	2010	2014	2019	*p*
*n*	200,502	232,229	214,140	213,087	208,450	
Sex						0.767
Boys	100,216 (50.0)	116,614 (50.2)	107,066 (50.0)	106,545 (50.0)	104,539 (50.2)	
Girls	100,286 (50.0)	115,615 (49.8)	107,074 (50.0)	106,542 (50.0)	103,911 (49.8)	
Age group						<0.001
7–12 y	99,490 (49.6)	115,051 (49.5)	106,988 (50.0)	106,651 (50.1)	105,296 (50.5)	
13–18 y	101,012 (50.4)	117,178 (50.5)	107,152 (50.0)	106,436 (49.9)	103,154 (49.5)	
Residence						0.043
Urban	99,211 (49.5)	116,915 (50.3)	107,031 (50.0)	106,647 (50.0)	104,237 (50.0)	
Rural	101,291 (50.5)	115,314 (49.7)	107,109 (50.0)	106,440 (50.0)	104,213 (50.0)	
Height (m)	1.49 (0.17)	1.50 (0.16)	1.51 (0.16)	1.52 (0.16)	1.53 (0.16)	<0.001
Weight (kg)	41.03 (14.20)	42.19 (14.26)	43.39 (14.51)	45.17 (15.13)	46.85 (16.25)	<0.001
BMI (kg/m^2^)	17.91 (3.50)	18.20 (3.26)	18.50 (3.35)	19.00 (3.58)	19.43 (3.91)	<0.001
SBP (mmHg)	103.95 (11.76)	102.86 (12.09)	104.18 (12.38)	104.83 (12.72)	107.23 (12.99)	<0.001
DBP (mmHg)	65.37 (9.16)	63.95 (9.64)	65.04 (9.53)	65.52 (9.49)	67.10 (9.49)	<0.001
OWOB_CN						<0.001
No	180,538 (90.0)	201,337 (86.7)	178,889 (83.5)	167,008 (78.4)	154,653 (74.2)	
Yes	19,964 (10.0)	30,892 (13.3)	35,251 (16.5)	46,079 (21.6)	53,797 (25.8)	
EBP						<0.001
No	182,321 (90.9)	216,474 (93.2)	195,005 (91.1)	194,687 (91.4)	182,925 (87.8)	
Yes	18,181 (9.1)	15,755 (6.8)	19,135 (8.9)	18,400 (8.6)	25,525 (12.2)	
Comorbidity_CN						<0.001
No	197,051 (98.3)	227,875 (98.1)	208,453 (97.3)	206,005 (96.7)	198,370 (95.2)	
Yes	3451 (1.7)	4354 (1.9)	5687 (2.7)	7082 (3.3)	10,080 (4.8)	
OWOB_US						<0.001
No	184,836 (92.2)	207,109 (89.2)	185,075 (86.4)	174,387 (81.8)	162,828 (78.1)	
Yes	15,666 (7.8)	25,120 (10.8)	29,065 (13.6)	38,700 (18.2)	45,622 (21.9)	
Comorbidity_US						<0.001
No	197,567 (98.5)	228,392 (98.3)	209,124 (97.7)	206,780 (97.0)	199,540 (95.7)	
Yes	2935 (1.5)	3837 (1.7)	5016 (2.3)	6307 (3.0)	8910 (4.3)	
HGS (kg)	20.06 (11.51)	22.64 (11.48)	22.89 (11.40)	22.95 (11.22)	22.50 (10.88)	<0.001
SBJ (m)	1.69 (0.37)	1.66 (0.37)	1.66 (0.38)	1.64 (0.38)	1.61 (0.38)	<0.001
MSI	1.60 (0.30)	1.62 (0.29)	1.61 (0.29)	1.57 (0.29)	1.52 (0.28)	<0.001

Data are presented as mean (standard deviation, SD) for continuous variables and frequency (percentage, %) for categorical variables. *p* values indicate the significance of differences or trends across the five survey waves, calculated using one-way analysis of variance (ANOVA) for continuous variables and Chi-square tests for categorical variables. Abbreviations: BMI, body mass index; SBP, systolic blood pressure; DBP, diastolic blood pressure; OWOB, overweight and obesity; EBP, elevated blood pressure. OWOB_CN: Overweight and obesity were defined according to the Screening for overweight and obesity among school-age children and adolescents (WS/T 586-2018) [19] released by the National Health Commission of the People’s Republic of China. OWOB_US: Overweight and obesity were defined based on the 2000 Centers for Disease Control and Prevention (CDC) growth charts, defined as BMI ≧ 85th percentile for sex and age. EBP: Elevated blood pressure was defined based on the Reference of screening for elevated blood pressure among children and adolescents (WS/T 610-2018) [20], defined as systolic and/or diastolic BP ≧ 95th percentile for sex, age, and height. Comorbidity_CN: Defined as the concurrent presence of both OWOB_CN and EBP. Comorbidity_US: Defined as the concurrent presence of both OWOB_US and EBP.

## Data Availability

The data presented in this study are available upon request from the corresponding author.

## Supplementary Materials

The following supporting information can be downloaded at: https://www.mdpi.com/article/10.3390/jcm15072712/s1, Figure S1: Flowchart of the study population selection process from the Chinese National Survey on Students’ Constitution and Health (CNSSCH, 2000–2019); Figure S2: Q–Q plots for HGS (A), SBJ (B), and MSI (C); Table S1: Intraclass correlation coefficients (ICCs) of cardiometabolic risks at the school level; Table S2: Temporal trends and future projections of OWOB, EBP, and comorbidity prevalence among Chinese children and adolescents (2000–2030); Figure S3: Sensitivity analysis: Temporal trends and future projections of OWOB, EBP, and comorbidity prevalence among Chinese children and adolescents (2000–2030); Table S3: Sensitivity analysis: Temporal trends and future projections of OWOB, EBP, and comorbidity prevalence among Chinese children and adolescents (2000–2030); Table S4: Temporal trends in handgrip strength, standing broad jump, and muscle strength index among Chinese children and adolescents (2000–2019); Figure S4: Sensitivity analysis: Dose–response associations of MSI with OWOB (US CDC 2000 definition), EBP, and their comorbidity; Figure S5: Sensitivity analysis: Dose–response associations of HGS/Wt and SBJ/Ht with OWOB, EBP, and their comorbidity; Table S5: Associations between categorical Muscle Strength Index (MSI) levels and cardiometabolic risks across five survey waves (2000–2019); Figure S6: Sensitivity analysis: Temporal consistency of the associations between MSI levels and cardiometabolic risks (defined by CDC 2000 growth charts); Figure S7: Sensitivity analysis: Temporal consistency of the associations between HGS/Wt levels and cardiometabolic risks; Figure S8: Sensitivity analysis: Temporal consistency of the associations between SBJ/Ht levels and cardiometabolic risks; Table S6: Sensitivity analysis: Associations between categorical MSI levels and cardiometabolic risks (OWOB defined by CDC 2000 growth charts); Table S7: Sensitivity analysis: Associations between categorical HGS/Wt, SBJ/Ht levels and cardiometabolic risks; Table S8: Normative reference values and LMS parameters for the Muscle Strength Index (MSI) in Chinese boys aged 7–18 years (based on 2019 CNSSCH data); Table S9: Normative reference values and LMS parameters for the Muscle Strength Index (MSI) in Chinese girls aged 7–18 years (based on 2019 CNSSCH data); Table S10: Population attributable fractions (PAFs) of cardiometabolic risks attributable to low muscular strength among Chinese children and adolescents by province (2019); Table S11: Sensitivity analysis: Population attributable fractions (PAFs) of cardiometabolic risks by province using CDC 2000 growth charts for OWOB definition (2019).

## Abbreviations

The following abbreviations are used in this manuscript:

BMI	Body Mass Index
CDC	Centers for Disease Control and Prevention
CI	Confidence Interval
CNSSCH	Chinese National Survey on Students’ Constitution and Health
DBP	Diastolic Blood Pressure
EBP	Elevated Blood Pressure
GLM	Generalized Linear Model
GLMM	Generalized Linear Mixed-Effects Model
HGS	Handgrip Strength
ICC	Intraclass Correlation Coefficient
LMS	Lambda–Mu–Sigma (method)
MSI	Muscle Strength Index
OR	Odds Ratio
OWOB	Overweight/Obesity
PAF	Population Attributable Fraction
RCS	Restricted Cubic Spline
SBJ	Standing Broad Jump
SBP	Systolic Blood Pressure
SD	Standard Deviation
SE	Standard Error
WHO	World Health Organization
