# Supplementary material for: Associations of the Muscle Strength Index with Overweight/Obesity, Elevated Blood Pressure, and Their Comorbidity in Chinese Children and Adolescents During Two Decades
Source: J Clin Med. 2026 Apr 3;15(7):2712. doi: 10.3390/jcm15072712 (PMC13073789; doi:10.3390/jcm15072712)
Supplement: Supplementary file 1 [file jcm-15-02712-s001.zip › jcm-4197832-supplementary.pdf]

Supplementary Materials

Figure S1. Flowchart of the study population selection process from the Chinese National Survey on Students' Constitution and Health (CNSSCH, 2000–2019).

Figure S2. Q–Q plots for HGS (A), SBJ (B), and MSI (C). Points represent the sample quantiles of each variable plotted against the theoretical quantiles of a normal distribution.

Table S1. Intraclass correlation coefficients (ICCs) of cardiometabolic risks at the school level.

Table S2. Temporal trends and future projections of OWOB, EBP, and comorbidity prevalence among Chinese children and adolescents (2000–2030).

Figure S3. Sensitivity analysis: Temporal trends and future projections of OWOB, EBP, and comorbidity prevalence among Chinese children and adolescents (2000–2030).

Table S3. Sensitivity analysis:Temporal trends and future projections of OWOB, EBP, and comorbidity prevalence among Chinese children and adolescents (2000–2030).

Table S4. Temporal trends in handgrip strength, standing broad jump, and muscle strength index among Chinese children and adolescents (2000–2019).

Figure S4. Sensitivity analysis: Dose–response associations of MSI with OWOB (US CDC 2000 definition), EBP, and their comorbidity.

Figure S5. Sensitivity analysis: Dose–response associations of HGS/Wt and SBJ/Ht with OWOB, EBP, and their comorbidity.

Table S5. Associations between categorical Muscle Strength Index (MSI) levels and cardiometabolic risks across five survey waves (2000–2019).

Figure S6. Sensitivity analysis: Temporal consistency of the associations between MSI levels and cardiometabolic risks (defined by CDC 2000 growth charts).

Figure S7. Sensitivity analysis: Temporal consistency of the associations between HGS/Wt levels and cardiometabolic risks.

Figure S8. Sensitivity analysis: Temporal consistency of the associations between SBJ/Ht levels and cardiometabolic risks.

Table S6. Sensitivity analysis: Associations between categorical MSI levels and cardiometabolic risks (OWOB defined by CDC 2000 growth charts).

Table S7. Sensitivity analysis: Associations between categorical HGS/Wt, SBJ/Ht levels and cardiometabolic risks.

Table S8. Normative reference values and LMS parameters for the Muscle Strength Index (MSI) in Chinese boys aged 7–18 years (based on 2019 CNSSCH data).

Table S9. Normative reference values and LMS parameters for the Muscle Strength Index (MSI) in Chinese girls aged 7–18 years (based on 2019 CNSSCH data).

Table S10. Population attributable fractions (PAFs) of cardiometabolic risks attributable to low muscular strength among Chinese children and adolescents by province (2019).

Table S11. Sensitivity analysis: Population attributable fractions (PAFs) of cardiometabolic risks by province using CDC 2000 growth charts for OWOB definition (2019).

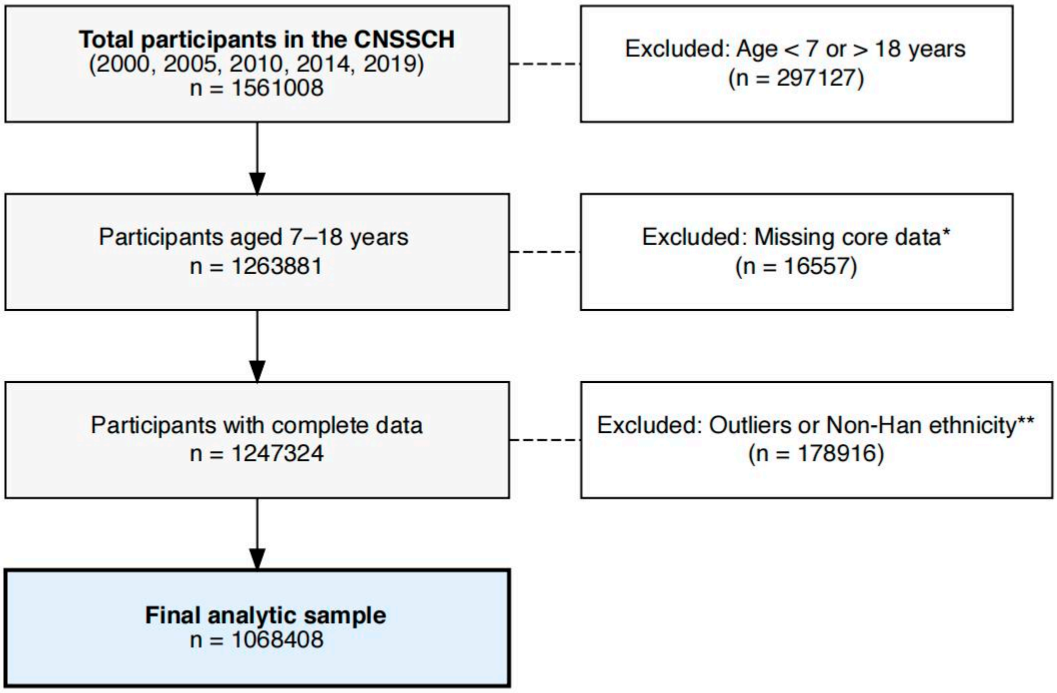

Figure S1. Flowchart of the study population selection process from the Chinese National Survey on Students' Constitution and Health (CNSSCH, 2000–2019).

Participants were excluded if they had missing data on key variables, biologically implausible values, or were not of Han ethnicity. The final analytical sample included 1,068,408 children and adolescents aged 7–18 years.

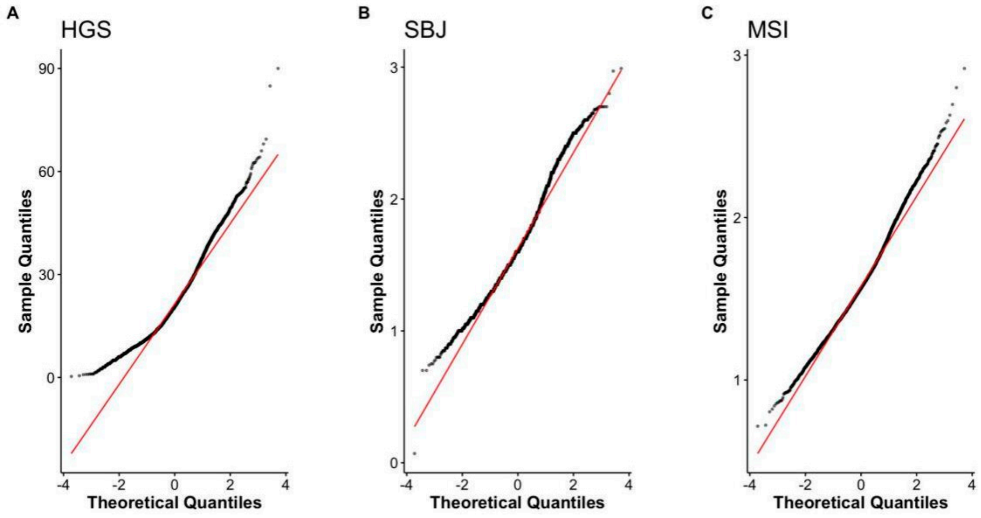

Figure S2. Q–Q plots for HGS (A), SBJ (B), and MSI (C). Points represent the sample quantiles of each variable plotted against the theoretical quantiles of a normal distribution.

The black dots represent the sample quantiles of each variable, while the red line indicates the theoretical quantiles of a normal distribution. Points closely following the red line suggest approximate normality. All three variables show roughly linear patterns with minor deviations at the tails, indicating that HGS, SBJ, and MSI are approximately normally distributed.

Table S1. Intraclass correlation coefficients (ICCs) of cardiometabolic risks at the school level.

Outcome	ICC
OWOB	0.103
EBP	0.265
Comorbidity	0.218

ICCs were calculated using null mixed-effects models to assess the clustering effect of outcomes within schools. An ICC > 0.10 indicates substantial clustering, justifying the use of generalized linear mixed-effects models (GLMMs) with school as a random effect. OWOB: overweight/obesity; EBP: elevated blood pressure.

Table S2. Temporal trends and future projections of OWOB, EBP, and comorbidity prevalence among Chinese children and adolescents (2000–2030).

year	OWOB	EBP	Comorbidity
2000*	9.96 (9.83–10.09)	9.07 (8.94–9.19)	1.72 (1.67–1.78)
2000	10.07 (9.95–10.19)	8.91 (8.77–9.04)	1.71 (1.66–1.76)
2001	10.57 (10.46–10.68)	8.48 (8.37–8.58)	1.74 (1.70–1.78)
2002	11.10 (10.99–11.21)	8.10 (8.00–8.18)	1.77 (1.73–1.81)
2003	11.66 (11.56–11.77)	7.77 (7.69–7.86)	1.81 (1.78–1.85)
2004	12.27 (12.17–12.39)	7.52 (7.43–7.60)	1.87 (1.83–1.91)
2005*	13.30 (13.16–13.44)	6.78 (6.68–6.89)	1.87 (1.82–1.93)
2005	12.93 (12.83–13.07)	7.35 (7.26–7.44)	1.93 (1.89–1.97)
2006	13.66 (13.56–13.78)	7.29 (7.20–7.37)	2.01 (1.97–2.06)
2007	14.45 (14.34–14.56)	7.32 (7.23–7.40)	2.12 (2.07–2.16)
2008	15.30 (15.20–15.40)	7.43 (7.35–7.51)	2.24 (2.19–2.28)
2009	16.19 (16.10–16.29)	7.62 (7.54–7.69)	2.39 (2.34–2.42)
2010*	16.46 (16.31–16.62)	8.94 (8.82–9.06)	2.66 (2.59–2.72)
2010	17.14 (17.02–17.24)	7.87 (7.79–7.94)	2.55 (2.50–2.59)
2011	18.11 (17.99–18.22)	8.19 (8.10–8.26)	2.73 (2.68–2.78)
2012	19.11 (18.96–19.24)	8.55 (8.46–8.63)	2.94 (2.89–2.98)
2013	20.12 (19.95–20.26)	8.97 (8.87–9.05)	3.16 (3.11–3.21)
2014*	21.62 (21.45–21.80)	8.63 (8.52–8.75)	3.32 (3.25–3.40)
2014	21.12 (20.94–21.25)	9.42 (9.32–9.51)	3.40 (3.34–3.46)
2015	22.09 (21.93–22.23)	9.89 (9.80–9.99)	3.65 (3.59–3.71)
2016	23.05 (22.91–23.18)	10.40 (10.31–10.49)	3.92 (3.86–3.97)
2017	24.01 (23.88–24.13)	10.93 (10.83–11.01)	4.20 (4.14–4.26)
2018	24.96 (24.82–25.09)	11.48 (11.35–11.57)	4.50 (4.44–4.58)
2019*	25.81 (25.62–26.00)	12.25 (12.11–12.39)	4.84 (4.74–4.93)
2019	25.92 (25.73–26.10)	12.06 (11.88–12.19)	4.83 (4.74–4.91)
2020	26.90 (26.64–27.12)	12.66 (12.44–12.85)	5.17 (5.05–5.29)
2021	27.91 (27.57–28.17)	13.29 (13.01–13.54)	5.54 (5.37–5.70)
2022	28.94 (28.53–29.28)	13.95 (13.60–14.26)	5.93 (5.72–6.14)

2023	29.99 (29.51–30.41)	14.64 (14.21–15.01)	6.34 (6.10–6.61)
2024	31.06 (30.50–31.57)	15.35 (14.84–15.79)	6.79 (6.50–7.12)
2025	32.16 (31.51–32.75)	16.09 (15.49–16.60)	7.26 (6.92–7.67)
2026	33.27 (32.54–33.96)	16.86 (16.17–17.45)	7.77 (7.38–8.26)
2027	34.40 (33.59–35.19)	17.65 (16.88–18.33)	8.30 (7.85–8.88)
2028	35.56 (34.65–36.43)	18.48 (17.61–19.26)	8.87 (8.36–9.56)
2029	36.72 (35.74–37.69)	19.34 (18.37–20.21)	9.47 (8.89–10.27)
2030	37.91 (36.83–38.97)	20.22 (19.15–21.21)	10.11 (9.46–11.03)

Data are presented as prevalence (%) with 95% confidence intervals (CIs). * indicate observed prevalence rates derived directly from the five national surveys (2000, 2005, 2010, 2014, and 2019). Values without * represent projected prevalence rates estimated using logistic regression models incorporating natural cubic splines (df=3) and bootstrap resampling. OWOB: overweight/obesity (defined by Chinese criteria, WS/T 586-2018); EBP: elevated blood pressure; Comorbidity: co-occurrence of OWOB and EBP.

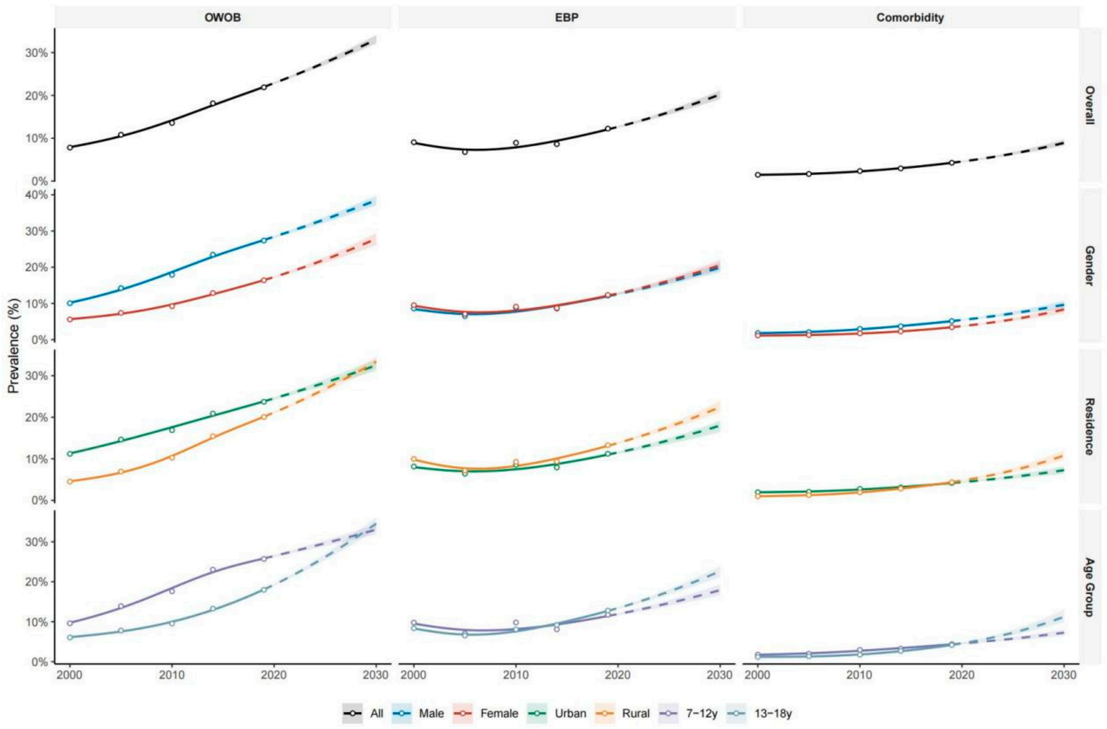

Figure S3. Sensitivity analysis: Temporal trends and future projections of OWOB, EBP, and comorbidity prevalence among Chinese children and adolescents (2000–2030).

The circles represent the crude observed prevalence rates derived from the five national surveys (2000–2019). The lines indicate the modeled trajectories estimated using logistic regression models incorporating natural cubic splines (df =3). Solid lines denote the fitted trends for the historical period (2000–2019), while dashed lines represent the projected trends for the future period (2020–2030). Shaded ribbons represent 95% confidence intervals derived from bootstrap resampling (100 iterations). Results are stratified by sex, residence, and age group.OWOB: overweight/obesity (defined by US CDC 2000 growth charts); EBP: elevated blood pressure; Comorbidity: co-occurrence of OWOB and EBP.

Table S3. Sensitivity analysis:Temporal trends and future projections of OWOB, EBP, and comorbidity prevalence among Chinese children and adolescents (2000–2030).

Data are presented as prevalence (%) with 95% confidence intervals (CIs). * indicate observed prevalence rates derived directly from the five national surveys (2000, 2005, 2010, 2014, and 2019). Values without * represent projected prevalence rates estimated using logistic regression models incorporating natural cubic splines (df=3) and bootstrap resampling. OWOB: overweight/obesity (defined by US CDC 2000 growth charts); EBP: elevated blood pressure; Comorbidity: co-occurrence of OWOB and EBP.

year	OWOB	EBP	Comorbidity
2000*	7.81 (7.70–7.93)	9.07 (8.94–9.19)	1.46 (1.41–1.52)
2000	7.92 (7.82–8.03)	8.91 (8.77–9.04)	1.46 (1.41–1.50)
2001	8.36 (8.28–8.47)	8.48 (8.37–8.58)	1.49 (1.45–1.53)
2002	8.84 (8.75–8.94)	8.10 (8.00–8.18)	1.53 (1.49–1.57)
2003	9.34 (9.26–9.45)	7.77 (7.69–7.86)	1.58 (1.54–1.61)
2004	9.89 (9.80–10.01)	7.52 (7.43–7.60)	1.63 (1.59–1.67)
2005*	10.82 (10.69–10.94)	6.78 (6.68–6.89)	1.65 (1.60–1.70)
2005	10.48 (10.39–10.60)	7.35 (7.26–7.44)	1.69 (1.65–1.74)
2006	11.13 (11.03–11.25)	7.29 (7.20–7.37)	1.77 (1.73–1.82)
2007	11.82 (11.73–11.94)	7.32 (7.23–7.40)	1.87 (1.83–1.91)
2008	12.57 (12.48–12.68)	7.43 (7.35–7.51)	1.98 (1.94–2.03)
2009	13.36 (13.27–13.45)	7.62 (7.54–7.69)	2.11 (2.07–2.15)
2010*	13.57 (13.43–13.72)	8.94 (8.82–9.06)	2.34 (2.28–2.41)
2010	14.19 (14.09–14.28)	7.87 (7.79–7.94)	2.26 (2.22–2.30)
2011	15.05 (14.94–15.15)	8.19 (8.10–8.26)	2.43 (2.38–2.46)
2012	15.93 (15.80–16.03)	8.55 (8.46–8.63)	2.61 (2.56–2.65)
2013	16.81 (16.67–16.92)	8.97 (8.87–9.05)	2.80 (2.75–2.85)
2014*	18.16 (18.00–18.33)	8.63 (8.52–8.75)	2.96 (2.89–3.03)
2014	17.70 (17.56–17.81)	9.42 (9.32–9.51)	3.01 (2.96–3.07)
2015	18.57 (18.43–18.67)	9.89 (9.80–9.99)	3.23 (3.18–3.30)
2016	19.42 (19.30–19.52)	10.40 (10.31–10.49)	3.47 (3.42–3.53)
2017	20.27 (20.15–20.37)	10.93 (10.83–11.01)	3.72 (3.66–3.77)
2018	21.12 (21.00–21.24)	11.48 (11.35–11.57)	3.98 (3.92–4.05)
2019*	21.89 (21.71–22.06)	12.25 (12.11–12.39)	4.27 (4.19–4.36)
2019	21.99 (21.83–22.14)	12.06 (11.88–12.19)	4.27 (4.17–4.36)
2020	22.87 (22.65–23.07)	12.66 (12.44–12.85)	4.57 (4.44–4.69)
2021	23.79 (23.50–24.03)	13.29 (13.01–13.54)	4.89 (4.73–5.05)
2022	24.73 (24.37–25.04)	13.95 (13.60–14.26)	5.24 (5.04–5.44)
2023	25.69 (25.27–26.08)	14.64 (14.21–15.01)	5.60 (5.36–5.86)

2024	26.67 (26.18–27.15)	15.35 (14.84–15.79)	5.99 (5.71–6.31)
2025	27.68 (27.12–28.24)	16.09 (15.49–16.60)	6.41 (6.08–6.79)
2026	28.72 (28.08–29.36)	16.86 (16.17–17.45)	6.85 (6.48–7.30)
2027	29.77 (29.06–30.51)	17.65 (16.88–18.33)	7.32 (6.89–7.85)
2028	30.85 (30.06–31.68)	18.48 (17.61–19.26)	7.82 (7.34–8.44)
2029	31.95 (31.07–32.87)	19.34 (18.37–20.21)	8.35 (7.79–9.07)
2030	33.07 (32.11–34.08)	20.22 (19.15–21.21)	8.92 (8.28–9.74)

Table S4. Temporal trends in handgrip strength, standing broad jump, and muscle strength index among Chinese children and adolescents (2000–2019).

Outcome	Group	2000	2005	2010	2014	2019
HGS (kg)	Overall	20.06 (20.01–20.11)	22.64 (22.59–22.69)	22.89 (22.84–22.94)	22.95 (22.90–23.00)	22.50 (22.45–22.55)
	Female	16.73 (16.68–16.78)	19.05 (19.01–19.09)	19.27 (19.22–19.31)	19.30 (19.26–19.34)	19.10 (19.06–19.14)
	Male	23.40 (23.32–23.48)	26.20 (26.12–26.28)	26.51 (26.43–26.59)	26.60 (26.52–26.68)	25.88 (25.80–25.95)
	13-18y	28.01 (27.94–28.07)	30.74 (30.69–30.80)	31.10 (31.04–31.16)	30.89 (30.83–30.95)	30.03 (29.98–30.09)
	7-12y	12.00 (11.96–12.03)	14.39 (14.36–14.42)	14.66 (14.63–14.69)	15.03 (14.99–15.06)	15.12 (15.08–15.15)
	Rural	20.13 (20.06–20.21)	22.83 (22.76–22.89)	22.93 (22.86–22.99)	23.15 (23.09–23.22)	22.64 (22.58–22.71)
	Urban	19.99 (19.92–20.06)	22.46 (22.39–22.52)	22.85 (22.78–22.92)	22.75 (22.68–22.81)	22.35 (22.29–22.42)
SBJ (m)	Overall	1.69 (1.69–1.69)	1.66 (1.66–1.66)	1.66 (1.66–1.67)	1.64 (1.63–1.64)	1.61 (1.61–1.61)
	Female	1.54 (1.54–1.55)	1.51 (1.51–1.51)	1.51 (1.51–1.51)	1.49 (1.49–1.49)	1.46 (1.46–1.47)
	Male	1.84 (1.84–1.84)	1.81 (1.81–1.81)	1.82 (1.81–1.82)	1.78 (1.78–1.78)	1.76 (1.76–1.76)
	13-18y	1.92 (1.92–1.92)	1.88 (1.88–1.89)	1.89 (1.89–1.89)	1.87 (1.87–1.87)	1.84 (1.84–1.85)
	7-12y	1.46 (1.46–1.46)	1.43 (1.43–1.44)	1.44 (1.44–1.44)	1.40 (1.40–1.40)	1.38 (1.38–1.39)
	Rural	1.69 (1.69–1.69)	1.66 (1.66–1.66)	1.66 (1.66–1.66)	1.64 (1.64–1.64)	1.61 (1.61–1.61)
	Urban	1.69 (1.69–1.69)	1.66 (1.66–1.66)	1.67 (1.67–1.67)	1.63 (1.63–1.63)	1.61 (1.61–1.62)
MSI	Overall	1.60 (1.60–1.60)	1.62 (1.62–1.62)	1.61 (1.61–1.61)	1.57 (1.57–1.57)	1.52 (1.52–1.52)
	Female	1.47 (1.46–1.47)	1.49 (1.49–1.49)	1.48 (1.48–1.48)	1.45 (1.45–1.45)	1.41 (1.41–1.41)
	Male	1.73 (1.73–1.73)	1.75 (1.75–1.76)	1.74 (1.74–1.74)	1.69 (1.68–1.69)	1.63 (1.63–1.64)
	13-18y	1.73 (1.73–1.73)	1.75 (1.75–1.75)	1.74 (1.74–1.74)	1.70 (1.70–1.70)	1.64 (1.64–1.64)
	7-12y	1.47 (1.46–1.47)	1.50 (1.50–1.50)	1.48 (1.48–1.48)	1.43 (1.43–1.44)	1.41 (1.40–1.41)
	Rural	1.63 (1.63–1.63)	1.66 (1.66–1.66)	1.63 (1.63–1.64)	1.59 (1.59–1.60)	1.54 (1.54–1.54)
	Urban	1.57 (1.56–1.57)	1.59 (1.59–1.59)	1.59 (1.58–1.59)	1.54 (1.54–1.54)	1.50 (1.50–1.51)

Data are presented as mean (95% confidence interval). HGS: handgrip strength; SBJ: standing broad jump; MSI: muscle strength index.

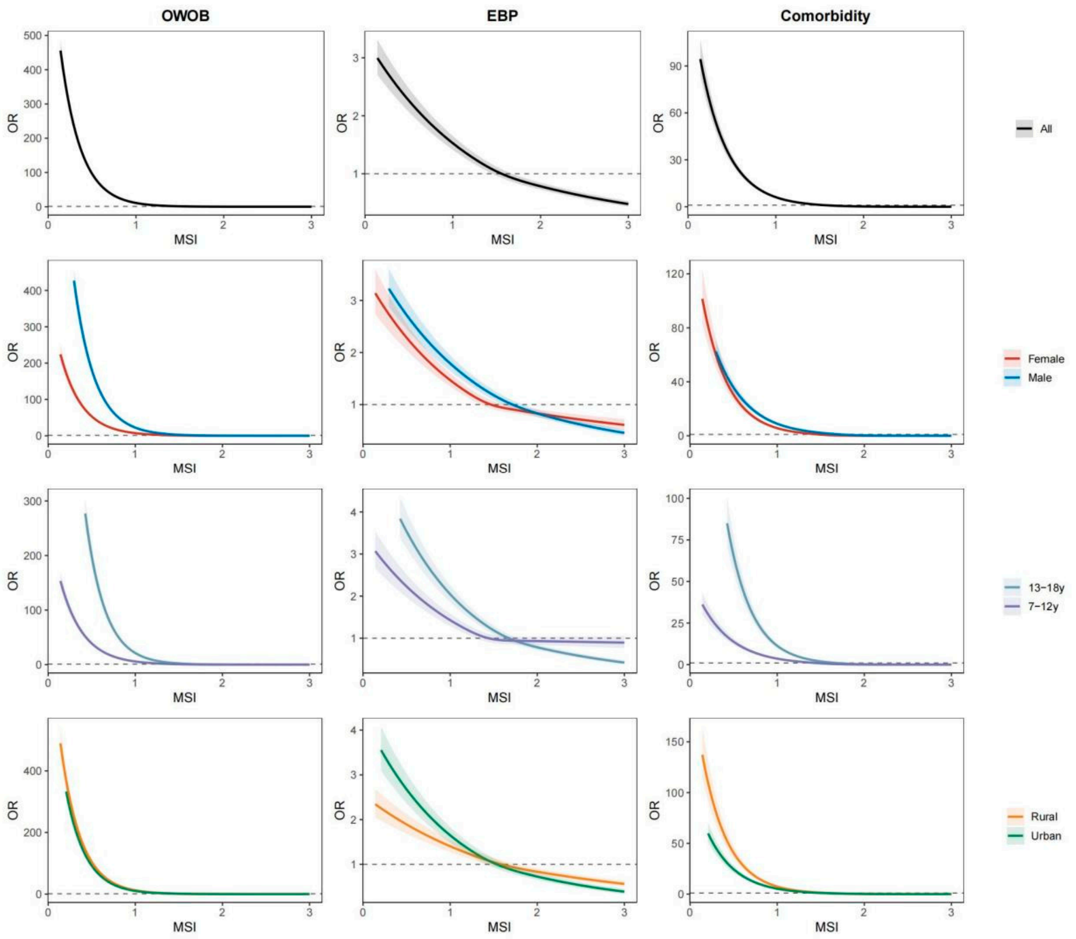

Figure S4. Sensitivity analysis: Dose–response associations of MSI with OWOB (US CDC 2000 definition), EBP, and their comorbidity.

Associations were estimated using generalized linear mixed-effects models (GLMMs) with restricted cubic splines (knots at 10th, 50th, 90th percentiles). The solid lines represent estimated odds ratios (ORs), and shaded ribbons indicate 95% CIs. OWOB: overweight/obesity (defined by US CDC 2000 growth charts); EBP: elevated blood pressure; Comorbidity: co-occurrence of OWOB and EBP.

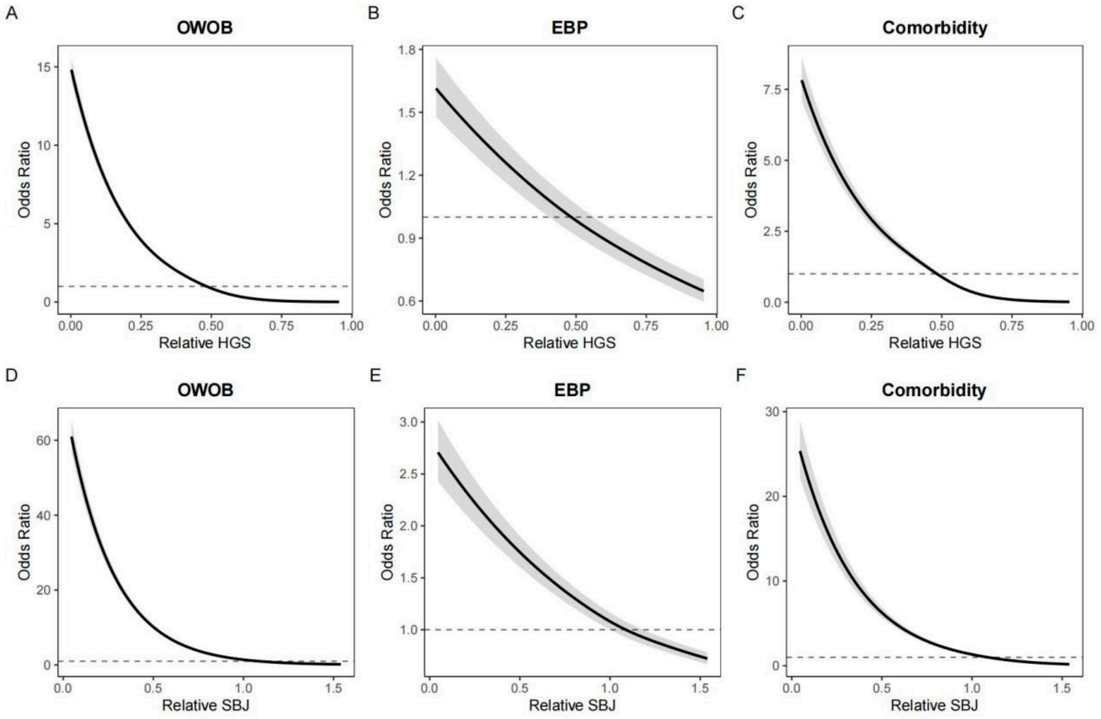

Figure S5. Sensitivity analysis: Dose–response associations of HGS/Wt and SBJ/Ht with OWOB, EBP, and their comorbidity.

Associations were estimated using generalized linear mixed-effects models (GLMMs) with restricted cubic splines (knots at 10th, 50th, 90th percentiles). The solid lines represent estimated odds ratios (ORs), and shaded ribbons indicate 95% CIs. OWOB: overweight/obesity; EBP: elevated blood pressure; Comorbidity: co-occurrence of OWOB and EBP; Wt: weight; Ht: height.

Table S5. Associations between categorical Muscle Strength Index (MSI) levels and cardiometabolic risks across five survey waves (2000–2019).

		OWOB		EBP		Comorbidity	
Year	MSI Level	OR	95%CI	OR	95%CI	OR	95%CI
2000	Low	3.02	(2.89 – 3.16)	1.06	(1.01 – 1.11)	2.70	(2.44 – 2.98)
	Mid-low	1.60	(1.53 – 1.67)	1.06	(1.01 – 1.11)	1.57	(1.41 – 1.75)
	Mid-high	0.63	(0.60 – 0.67)	0.97	(0.93 – 1.02)	0.63	(0.55 – 0.72)
	High	0.40	(0.37 – 0.42)	0.98	(0.94 – 1.03)	0.38	(0.33 – 0.45)
2005	Low	4.15	(4.00 – 4.31)	1.59	(1.51 – 1.68)	3.94	(3.61 – 4.31)
	Mid-low	1.84	(1.78 – 1.91)	1.19	(1.13 – 1.26)	1.91	(1.74 – 2.11)
	Mid-high	0.54	(0.52 – 0.57)	0.96	(0.92 – 1.01)	0.55	(0.49 – 0.62)
	High	0.26	(0.25 – 0.28)	0.92	(0.87 – 0.96)	0.27	(0.23 – 0.31)
2010	Low	4.21	(4.06 – 4.36)	1.46	(1.40 – 1.54)	3.55	(3.29 – 3.85)
	Mid-low	1.88	(1.81 – 1.95)	1.17	(1.12 – 1.23)	1.85	(1.70 – 2.02)
	Mid-high	0.56	(0.53 – 0.58)	0.96	(0.92 – 1.01)	0.62	(0.56 – 0.69)
	High	0.25	(0.24 – 0.27)	0.95	(0.91 – 1.00)	0.29	(0.25 – 0.33)
2014	Low	4.57	(4.42 – 4.72)	1.55	(1.48 – 1.62)	3.44	(3.20 – 3.69)
	Mid-low	1.90	(1.84 – 1.97)	1.21	(1.15 – 1.27)	1.72	(1.59 – 1.86)
	Mid-high	0.57	(0.55 – 0.60)	0.93	(0.88 – 0.98)	0.55	(0.50 – 0.61)
	High	0.28	(0.26 – 0.29)	0.94	(0.89 – 0.99)	0.30	(0.27 – 0.35)
2019	Low	4.81	(4.66 – 4.97)	1.42	(1.37 – 1.49)	3.50	(3.27 – 3.75)
	Mid-low	1.82	(1.76 – 1.88)	1.13	(1.08 – 1.18)	1.65	(1.53 – 1.78)
	Mid-high	0.57	(0.54 – 0.60)	0.99	(0.94 – 1.04)	0.67	(0.60 – 0.74)
	High	0.31	(0.29 – 0.33)	0.98	(0.93 – 1.04)	0.35	(0.31 – 0.40)

Data correspond to Figure 4 in the main text. Values are adjusted Odds Ratios (ORs) with 95% confidence intervals (CIs) estimated from generalized linear mixed-effects models (GLMMs). Models were adjusted for age group, sex, and residence, with school included as a random effect. The "Mid" group (40th–60th percentile) served as the reference category. OWOB: overweight/obesity (Chinese criteria); EBP: elevated blood pressure; Comorbidity: co-occurrence of OWOB and EBP.

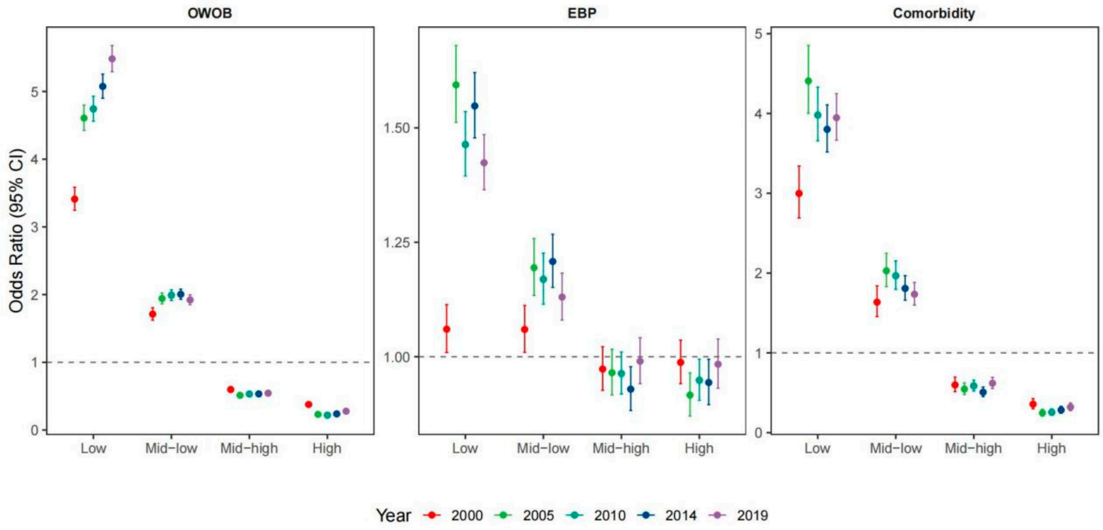

Figure S6. Sensitivity analysis: Temporal consistency of the associations between MSI levels and cardiometabolic risks (defined by CDC 2000 growth charts).

This figure corresponds to Figure 4 in the main text but uses the US CDC 2000 growth charts to define overweight/obesity. Data are presented as odds ratios (ORs) with 95% confidence intervals (CIs). Separate GLMMs were fitted for each survey year. MSI was categorized into five levels based on sex- and age-specific percentiles: Low (<20th), Mid-low (20th–40th), Mid (40th–60th, Reference group), Mid-high (60th–80th), and High (≥80th). OWOB: overweight/obesity defined by CDC 2000 criteria; EBP: elevated blood pressure; Comorbidity: co-occurrence of OWOB and EBP.

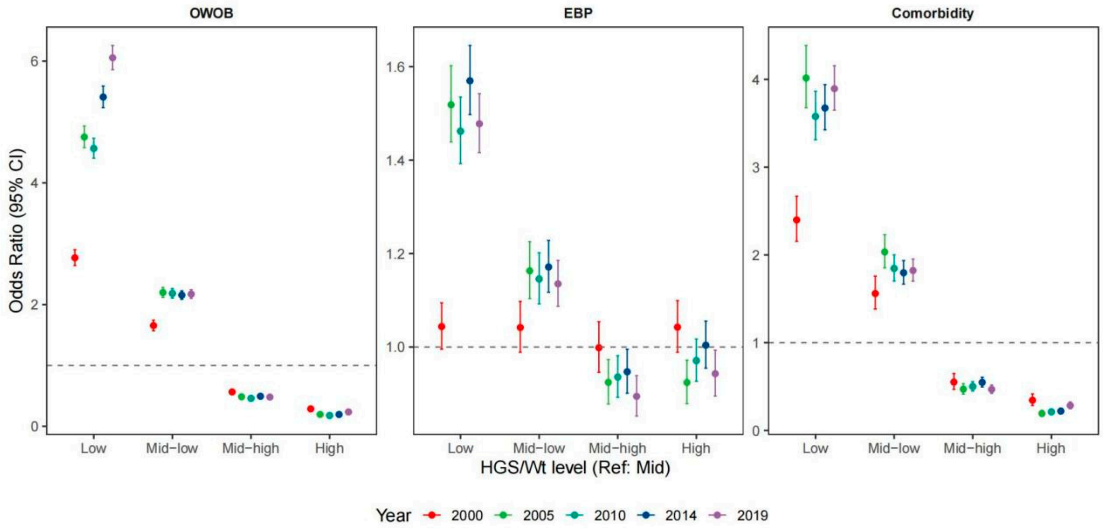

Figure S7. Sensitivity analysis: Temporal consistency of the associations between HGS/Wt levels and cardiometabolic risks.

Data are presented as odds ratios (ORs) with 95% confidence intervals (CIs). Separate GLMMs were fitted for each survey year to assess the stability of associations over time. MSI was categorized into five levels based on sex- and age-specific percentiles: Low (<20th), Mid-low (20th–40th), Mid (40th–60th, Reference group), Mid-high (60th–80th), and High (≥80th). The dashed vertical line indicates an OR of 1.0. OWOB: overweight/obesity; EBP: elevated blood pressure; Wt: weight.

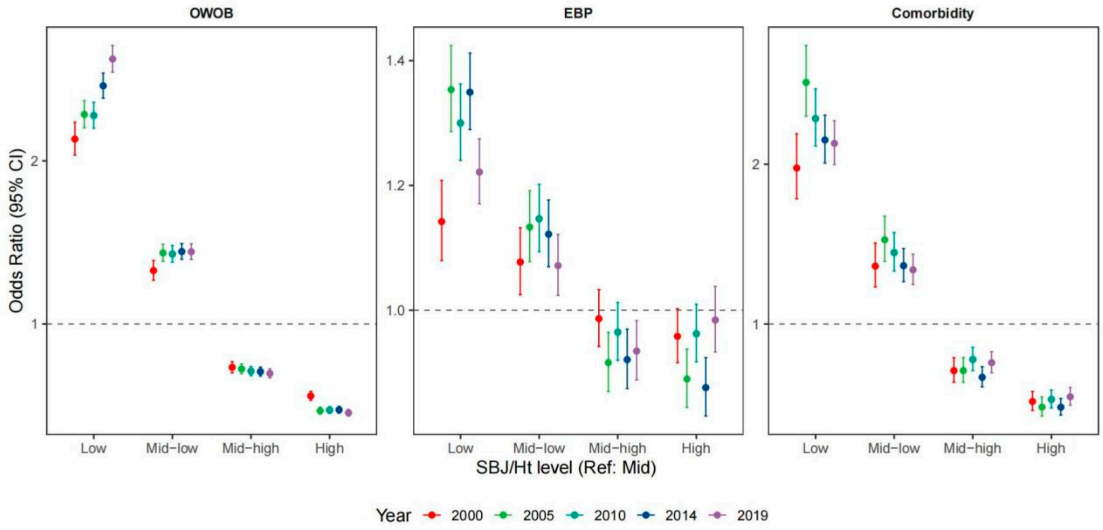

Figure S8. Sensitivity analysis: Temporal consistency of the associations between SBJ/Ht levels and cardiometabolic risks.

Data are presented as odds ratios (ORs) with 95% confidence intervals (CIs). Separate GLMMs were fitted for each survey year to assess the stability of associations over time. MSI was categorized into five levels based on sex- and age-specific percentiles: Low (<20th), Mid-low (20th–40th), Mid (40th–60th, Reference group), Mid-high (60th–80th), and High (≥80th). The dashed vertical line indicates an OR of 1.0. OWOB: overweight/obesity; EBP: elevated blood pressure; Ht: height.

Table S6. Sensitivity analysis: Associations between categorical MSI levels and cardiometabolic risks (OWOB defined by CDC 2000 growth charts).

		OWOB		EBP		Comorbidity	
Year	MSI Level	OR	95%CI	OR	95%CI	OR	95%CI
2000	Low	3.41	(3.25 – 3.58)	1.06	(1.01 – 1.11)	3.00	(2.69 – 3.34)
	Mid-low	1.71	(1.62 – 1.80)	1.06	(1.01 – 1.11)	1.64	(1.45 – 1.84)
	Mid-high	0.60	(0.56 – 0.64)	0.97	(0.93 – 1.02)	0.60	(0.51 – 0.70)
	High	0.38	(0.35 – 0.41)	0.99	(0.94 – 1.04)	0.36	(0.30 – 0.43)
2005	Low	4.61	(4.42 – 4.80)	1.59	(1.51 – 1.68)	4.41	(4.00 – 4.85)
	Mid-low	1.94	(1.86 – 2.02)	1.19	(1.13 – 1.26)	2.03	(1.83 – 2.25)
	Mid-high	0.51	(0.49 – 0.54)	0.97	(0.92 – 1.02)	0.55	(0.48 – 0.62)
	High	0.23	(0.22 – 0.25)	0.92	(0.87 – 0.96)	0.25	(0.21 – 0.29)
2010	Low	4.74	(4.56 – 4.93)	1.46	(1.40 – 1.54)	3.98	(3.66 – 4.33)
	Mid-low	1.99	(1.91 – 2.07)	1.17	(1.11 – 1.23)	1.97	(1.80 – 2.15)
	Mid-high	0.53	(0.51 – 0.56)	0.96	(0.92 – 1.01)	0.59	(0.52 – 0.66)
	High	0.22	(0.21 – 0.23)	0.95	(0.90 – 0.99)	0.26	(0.22 – 0.30)
2014	Low	5.07	(4.90 – 5.26)	1.55	(1.48 – 1.62)	3.80	(3.52 – 4.11)
	Mid-low	2.00	(1.93 – 2.08)	1.21	(1.15 – 1.27)	1.81	(1.66 – 1.97)
	Mid-high	0.53	(0.51 – 0.56)	0.93	(0.88 – 0.98)	0.51	(0.45 – 0.57)
	High	0.24	(0.22 – 0.25)	0.94	(0.90 – 0.99)	0.28	(0.24 – 0.33)
2019	Low	5.48	(5.29 – 5.68)	1.42	(1.36 – 1.49)	3.95	(3.67 – 4.25)
	Mid-low	1.92	(1.85 – 2.00)	1.13	(1.08 – 1.18)	1.73	(1.60 – 1.88)
	Mid-high	0.54	(0.52 – 0.57)	0.99	(0.94 – 1.04)	0.62	(0.55 – 0.69)
	High	0.28	(0.26 – 0.30)	0.98	(0.93 – 1.04)	0.32	(0.28 – 0.37)

Data correspond to Appendix Figure S5. Values are adjusted Odds Ratios (ORs) with 95% confidence intervals (CIs). Values are adjusted Odds Ratios (ORs) with 95% confidence intervals (CIs) estimated from generalized linear mixed-effects models (GLMMs). Models were adjusted for age group, sex, and residence, with school included as a random effect. The "Mid" group (40th–60th percentile) served as the reference category. OWOB: overweight/obesity (defined by CDC 2000 growth charts); EBP: elevated blood pressure; Comorbidity: co-occurrence of OWOB and EBP.

Table S7. Sensitivity analysis: Associations between categorical HGS/Wt, SBJ/Ht levels and cardiometabolic risks.

Year	Strength Level	OWOB	EBP	Comorbidity
		HGS/Wt		
2000	Low	2.77 (2.64 – 2.90)	1.04 (1.00 – 1.09)	2.40 (2.16 – 2.67)
	Mid-low	1.66 (1.57 – 1.74)	1.04 (0.99 – 1.10)	1.56 (1.38 – 1.76)
	Mid-high	0.56 (0.53 – 0.60)	1.00 (0.95 – 1.05)	0.55 (0.47 – 0.65)
	High	0.29 (0.26 – 0.31)	1.04 (0.99 – 1.10)	0.34 (0.28 – 0.42)
2005	Low	4.75 (4.58 – 4.93)	1.52 (1.44 – 1.60)	4.02 (3.68 – 4.39)
	Mid-low	2.20 (2.12 – 2.28)	1.16 (1.10 – 1.23)	2.03 (1.85 – 2.23)
	Mid-high	0.49 (0.46 – 0.51)	0.92 (0.88 – 0.97)	0.47 (0.41 – 0.53)
	High	0.19 (0.18 – 0.21)	0.92 (0.88 – 0.97)	0.19 (0.16 – 0.23)
2010	Low	4.57 (4.41 – 4.73)	1.46 (1.39 – 1.54)	3.58 (3.31 – 3.86)
	Mid-low	2.18 (2.11 – 2.26)	1.15 (1.09 – 1.20)	1.85 (1.70 – 2.00)
	Mid-high	0.46 (0.44 – 0.48)	0.94 (0.89 – 0.98)	0.50 (0.45 – 0.56)
	High	0.18 (0.17 – 0.19)	0.97 (0.93 – 1.02)	0.21 (0.18 – 0.24)
2014	Low	5.41 (5.24 – 5.59)	1.57 (1.50 – 1.65)	3.67 (3.43 – 3.94)
	Mid-low	2.16 (2.09 – 2.23)	1.17 (1.12 – 1.23)	1.80 (1.67 – 1.94)
	Mid-high	0.49 (0.47 – 0.51)	0.95 (0.90 – 1.00)	0.55 (0.50 – 0.61)
	High	0.19 (0.18 – 0.21)	1.00 (0.95 – 1.06)	0.22 (0.19 – 0.25)
2019	Low	6.05 (5.86 – 6.26)	1.48 (1.42 – 1.54)	3.89 (3.65 – 4.16)
	Mid-low	2.17 (2.10 – 2.25)	1.14 (1.09 – 1.19)	1.82 (1.70 – 1.95)
	Mid-high	0.48 (0.46 – 0.50)	0.89 (0.85 – 0.94)	0.47 (0.42 – 0.52)
	High	0.24 (0.22 – 0.25)	0.94 (0.90 – 0.99)	0.28 (0.25 – 0.32)
		SBJ/Ht		
2000	Low	2.13 (2.04 – 2.24)	1.14 (1.08 – 1.21)	1.98 (1.78 – 2.19)
	Mid-low	1.33 (1.27 – 1.39)	1.08 (1.02 – 1.13)	1.36 (1.23 – 1.51)
	Mid-high	0.73 (0.70 – 0.77)	0.99 (0.94 – 1.03)	0.71 (0.64 – 0.79)
	High	0.56 (0.53 – 0.59)	0.96 (0.92 – 1.00)	0.52 (0.46 – 0.58)
2005	Low	2.28 (2.20 – 2.37)	1.35 (1.29 – 1.42)	2.51 (2.30 – 2.74)
	Mid-low	1.44 (1.38 – 1.49)	1.13 (1.08 – 1.19)	1.53 (1.39 – 1.67)
	Mid-high	0.72 (0.70 – 0.75)	0.92 (0.87 – 0.96)	0.71 (0.64 – 0.79)
	High	0.47 (0.45 – 0.49)	0.89 (0.84 – 0.94)	0.48 (0.42 – 0.54)
2010	Low	2.28 (2.20 – 2.36)	1.30 (1.24 – 1.36)	2.29 (2.11 – 2.47)
	Mid-low	1.43 (1.38 – 1.48)	1.15 (1.09 – 1.20)	1.45 (1.33 – 1.57)
	Mid-high	0.71 (0.68 – 0.74)	0.96 (0.92 – 1.01)	0.78 (0.71 – 0.86)
	High	0.47 (0.45 – 0.49)	0.96 (0.92 – 1.01)	0.53 (0.48 – 0.59)
2014	Low	2.46 (2.38 – 2.54)	1.35 (1.29 – 1.41)	2.15 (2.01 – 2.31)
	Mid-low	1.44 (1.40 – 1.49)	1.12 (1.07 – 1.18)	1.36 (1.27 – 1.47)
	Mid-high	0.71 (0.68 – 0.74)	0.92 (0.87 – 0.97)	0.67 (0.61 – 0.73)

	High	0.47 (0.45 – 0.50)	0.88 (0.83 – 0.92)	0.48 (0.43 – 0.53)
2019	Low	2.62 (2.54 – 2.71)	1.22 (1.17 – 1.27)	2.13 (2.00 – 2.27)
	Mid-low	1.44 (1.40 – 1.49)	1.07 (1.02 – 1.12)	1.34 (1.25 – 1.44)
	Mid-high	0.70 (0.67 – 0.72)	0.93 (0.89 – 0.98)	0.76 (0.70 – 0.83)
	High	0.46 (0.44 – 0.48)	0.98 (0.93 – 1.04)	0.54 (0.49 – 0.60)

Data correspond to Appendix Figure S7-S8. Values are adjusted Odds Ratios (ORs) with 95% confidence intervals (CIs). Values are adjusted Odds Ratios (ORs) with 95% confidence intervals (CIs) estimated from generalized linear mixed-effects models (GLMMs). Models were adjusted for age group, sex, and residence, with school included as a random effect. The "Mid" group (40th–60th percentile) served as the reference category. OWOB: overweight/obesity (defined by CDC 2000 growth charts); EBP: elevated blood pressure; Comorbidity: co-occurrence of OWOB and EBP.

Table S8. Normative reference values and LMS parameters for the Muscle Strength Index (MSI) in Chinese boys aged 7–18 years (based on 2019 CNSSCH data).

Age	P3	P5	P10	P15	P25	P30	P40	P50	P60	P70	P75	P85	P90	P95	P97
7.0	0.97	1.02	1.10	1.15	1.22	1.25	1.30	1.36	1.41	1.46	1.50	1.58	1.63	1.72	1.78
7.5	0.99	1.04	1.12	1.17	1.24	1.27	1.33	1.38	1.43	1.49	1.52	1.60	1.66	1.75	1.82
8.0	1.00	1.05	1.13	1.18	1.26	1.29	1.34	1.40	1.45	1.51	1.54	1.63	1.68	1.78	1.84
8.5	1.01	1.07	1.14	1.19	1.27	1.30	1.36	1.41	1.47	1.53	1.56	1.64	1.70	1.79	1.86
9.0	1.02	1.07	1.15	1.21	1.28	1.31	1.37	1.43	1.48	1.54	1.57	1.66	1.72	1.81	1.87
9.5	1.03	1.08	1.16	1.22	1.29	1.33	1.38	1.44	1.50	1.56	1.59	1.67	1.73	1.82	1.89
10.0	1.04	1.09	1.17	1.23	1.31	1.34	1.40	1.46	1.51	1.58	1.61	1.69	1.75	1.84	1.91
10.5	1.05	1.10	1.19	1.25	1.33	1.36	1.42	1.48	1.54	1.60	1.63	1.72	1.78	1.87	1.93
11.0	1.06	1.12	1.21	1.27	1.35	1.39	1.45	1.51	1.56	1.63	1.66	1.75	1.81	1.90	1.96
11.5	1.09	1.15	1.24	1.30	1.38	1.42	1.48	1.54	1.60	1.66	1.70	1.78	1.84	1.93	1.99
12.0	1.11	1.18	1.27	1.33	1.42	1.46	1.52	1.58	1.64	1.70	1.74	1.82	1.88	1.97	2.03
12.5	1.15	1.21	1.31	1.37	1.46	1.50	1.56	1.62	1.68	1.74	1.78	1.86	1.92	2.01	2.07
13.0	1.19	1.25	1.35	1.41	1.50	1.54	1.60	1.66	1.72	1.79	1.82	1.91	1.97	2.05	2.11
13.5	1.23	1.29	1.39	1.45	1.54	1.58	1.65	1.71	1.77	1.83	1.86	1.95	2.01	2.09	2.15
14.0	1.27	1.33	1.43	1.49	1.58	1.62	1.68	1.75	1.80	1.87	1.90	1.99	2.04	2.13	2.19
14.5	1.30	1.37	1.46	1.53	1.62	1.66	1.72	1.78	1.84	1.90	1.94	2.02	2.08	2.16	2.22
15.0	1.33	1.40	1.50	1.56	1.65	1.69	1.75	1.81	1.87	1.93	1.97	2.05	2.11	2.19	2.24
15.5	1.36	1.43	1.53	1.59	1.68	1.72	1.78	1.84	1.90	1.96	2.00	2.08	2.13	2.21	2.27
16.0	1.38	1.45	1.55	1.61	1.70	1.74	1.81	1.86	1.92	1.98	2.02	2.10	2.15	2.23	2.28
16.5	1.40	1.47	1.57	1.63	1.73	1.76	1.83	1.89	1.94	2.00	2.04	2.12	2.17	2.25	2.30
17.0	1.42	1.49	1.59	1.65	1.74	1.78	1.85	1.90	1.96	2.02	2.05	2.13	2.18	2.26	2.31
17.5	1.44	1.51	1.61	1.67	1.76	1.80	1.86	1.92	1.98	2.04	2.07	2.15	2.20	2.27	2.32
18.0	1.45	1.52	1.62	1.69	1.78	1.81	1.88	1.94	1.99	2.05	2.08	2.16	2.21	2.28	2.33

The values were derived using the Lambda-Mu-Sigma (LMS) method with a Box-Cox t-distribution (BCT). L: Box-Cox power (skewness); M: median; S: coefficient of variation. P3–P97 represent the 3rd to 97th percentiles.

Table S9. Normative reference values and LMS parameters for the Muscle Strength Index (MSI) in Chinese girls aged 7–18 years (based on 2019 CNSSCH data).

Age	P3	P5	P10	P15	P25	P30	P40	P50	P60	P70	P75	P85	P90	P95	P97
7.0	0.92	0.97	1.04	1.09	1.15	1.18	1.23	1.28	1.33	1.38	1.41	1.48	1.53	1.62	1.68
7.5	0.94	0.99	1.06	1.11	1.18	1.20	1.26	1.30	1.35	1.40	1.43	1.51	1.56	1.65	1.71
8.0	0.96	1.01	1.08	1.13	1.20	1.23	1.28	1.32	1.37	1.42	1.45	1.53	1.58	1.67	1.73
8.5	0.98	1.03	1.10	1.15	1.21	1.24	1.29	1.34	1.39	1.44	1.47	1.55	1.60	1.69	1.75
9.0	0.99	1.04	1.11	1.16	1.23	1.26	1.31	1.36	1.40	1.46	1.49	1.56	1.62	1.70	1.76
9.5	1.00	1.05	1.12	1.17	1.24	1.27	1.32	1.37	1.42	1.47	1.50	1.58	1.63	1.71	1.77
10.0	1.01	1.06	1.13	1.18	1.25	1.28	1.33	1.38	1.43	1.48	1.51	1.59	1.64	1.72	1.78
10.5	1.02	1.07	1.14	1.19	1.26	1.29	1.34	1.39	1.43	1.49	1.52	1.59	1.65	1.73	1.79
11.0	1.02	1.07	1.15	1.19	1.26	1.29	1.34	1.39	1.44	1.49	1.52	1.60	1.65	1.74	1.80
11.5	1.03	1.08	1.15	1.20	1.27	1.30	1.35	1.40	1.45	1.50	1.53	1.60	1.66	1.74	1.80
12.0	1.04	1.09	1.16	1.21	1.28	1.31	1.36	1.40	1.45	1.51	1.53	1.61	1.66	1.74	1.80
12.5	1.05	1.10	1.17	1.22	1.28	1.31	1.36	1.41	1.46	1.51	1.54	1.61	1.66	1.74	1.80
13.0	1.06	1.10	1.18	1.22	1.29	1.32	1.37	1.42	1.46	1.52	1.55	1.62	1.67	1.75	1.80
13.5	1.06	1.11	1.18	1.23	1.30	1.33	1.38	1.42	1.47	1.52	1.55	1.62	1.67	1.75	1.80
14.0	1.07	1.12	1.19	1.24	1.31	1.34	1.39	1.43	1.48	1.53	1.56	1.63	1.68	1.75	1.80
14.5	1.08	1.13	1.20	1.25	1.32	1.34	1.39	1.44	1.49	1.54	1.57	1.64	1.68	1.76	1.81
15.0	1.09	1.14	1.21	1.26	1.32	1.35	1.40	1.45	1.50	1.55	1.57	1.64	1.69	1.77	1.82
15.5	1.10	1.15	1.22	1.27	1.33	1.36	1.41	1.46	1.50	1.55	1.58	1.65	1.70	1.77	1.82
16.0	1.11	1.16	1.23	1.27	1.34	1.37	1.42	1.46	1.51	1.56	1.59	1.66	1.71	1.78	1.83
16.5	1.11	1.16	1.23	1.28	1.35	1.38	1.42	1.47	1.52	1.57	1.60	1.66	1.71	1.79	1.84
17.0	1.12	1.17	1.24	1.29	1.35	1.38	1.43	1.48	1.52	1.57	1.60	1.67	1.72	1.79	1.84
17.5	1.13	1.17	1.25	1.29	1.36	1.39	1.44	1.48	1.53	1.58	1.61	1.68	1.73	1.80	1.85
18.0	1.13	1.18	1.25	1.30	1.37	1.39	1.44	1.49	1.54	1.59	1.61	1.68	1.73	1.81	1.86

The values were derived using the Lambda-Mu-Sigma (LMS) method with a Box-Cox t-distribution (BCT). L: Box-Cox power (skewness); M: median; S: coefficient of variation. P3–P97 represent the 3rd to 97th percentiles.

Table S10. Population attributable fractions (PAFs) of cardiometabolic risks attributable to low muscular strength among Chinese children and adolescents by province (2019).

Region	OWOB	EBP	Comorbidity
National	43.5 (42.9 - 44.1)	12.3 (11.2 - 13.2)	48.1 (46.6 - 49.6)
Beijing	40.8 (37.8 - 44.3)	14.4 (6.9 - 23.6)	44.1 (35.7 - 53.2)
Tianjin	49.9 (45.9 - 54.5)	26.5 (20.8 - 32.9)	59.1 (51.2 - 66.2)
Hebei	36.6 (30.6 - 42.6)	11.6 (5.5 - 19.3)	39.9 (25.2 - 51.9)
Shanxi	43.0 (38.8 - 47.0)	13.8 (9.3 - 19.9)	44.1 (34.9 - 51.9)
Inner Mongolia	50.0 (44.3 - 54.5)	20.7 (15.7 - 26.0)	59.1 (50.8 - 64.5)
Liaoning	51.3 (45.1 - 56.2)	11.4 (8.1 - 14.5)	55.5 (49.8 - 62.0)
Jilin	49.2 (43.9 - 55.2)	4.5 (-7.8 - 16.4)	46.7 (33.8 - 57.7)
Heilongjiang	54.9 (52.2 - 59.1)	14.5 (5.9 - 23.3)	62.3 (49.2 - 72.6)
Shanghai	49.4 (45.3 - 53.6)	5.9 (0.9 - 10.6)	45.2 (37.3 - 51.6)
Jiangsu	31.2 (26.9 - 36.2)	4.7 (-1.7 - 10.9)	33.6 (25.8 - 47.7)
Zhejiang	32.3 (27.7 - 38.0)	8.5 (2.3 - 13.4)	30.8 (21.0 - 42.2)
Anhui	43.9 (40.9 - 47.1)	8.5 (3.6 - 13.1)	50.6 (44.7 - 55.8)
Fujian	45.3 (41.1 - 49.1)	16.5 (8.9 - 24.1)	60.7 (52.7 - 71.9)
Jiangxi	39.3 (35.5 - 44.7)	10.6 (2.6 - 17.6)	47.6 (37.0 - 56.9)
Shandong	42.4 (39.2 - 47.0)	13.8 (7.1 - 21.2)	48.5 (39.5 - 59.2)
Henan	47.2 (43.1 - 51.9)	7.7 (-7.2 - 17.7)	41.1 (30.6 - 50.7)
Hubei	44.2 (40.0 - 51.1)	6.7 (2.7 - 13.2)	44.5 (36.9 - 51.6)
Hunan	33.0 (29.3 - 36.8)	6.1 (-0.6 - 12.7)	40.8 (28.8 - 51.7)
Guangdong	41.4 (36.3 - 45.9)	10.9 (7.3 - 17.0)	41.0 (32.3 - 49.7)
Guangxi	45.9 (40.5 - 51.0)	12.5 (5.2 - 21.8)	46.6 (34.8 - 64.4)
Hainan	40.3 (33.4 - 45.9)	1.7 (-1.2 - 5.6)	30.3 (22.2 - 39.4)
Chongqing	34.6 (30.7 - 38.2)	4.6 (-2.1 - 16.8)	41.7 (23.6 - 55.9)
Sichuan	43.8 (39.0 - 48.4)	10.8 (4.0 - 17)	48.3 (35.2 - 62.8)
Guizhou	45.5 (40.3 - 50.4)	4.7 (-0.9 - 11.6)	45.1 (36.5 - 53.3)
Yunnan	45.3 (40.9 - 49.4)	6.7 (1.3 - 14.0)	51.7 (36.9 - 61.8)
Shaanxi	54.3 (49.5 - 60.4)	15.1 (4.9 - 31.5)	61.1 (47.9 - 73.7)
Gansu	44.3 (40.5 - 49.4)	9.6 (5.5 - 14.4)	48.3 (41.1 - 60.2)
Qinghai	45.1 (35.1 - 52.5)	6.7 (3.2 - 9.5)	52.6 (45.3 - 58.6)
Ningxia	43.4 (35.3 - 50.1)	-0.7 (-9.4 - 6.6)	36.0 (23.0 - 44.1)
Xinjiang	43.5 (36.2 - 48.8)	3.8 (-4.9 - 15.4)	41.0 (30.3 - 53.9)

Data correspond to Figure 6 in the main text. The PAFs estimate the percentage of cases (OWOB, EBP, and Comorbidity) that could theoretically be prevented if children with low muscular strength (MSI <40th percentile) improved their strength to at least the moderate level (≥40th percentile). OWOB: overweight/obesity (Chinese criteria); EBP: elevated blood pressure; Comorbidity: co-occurrence of OWOB and EBP.

Table S11. Sensitivity analysis: Population attributable fractions (PAFs) of cardiometabolic risks by province using CDC 2000 growth charts for OWOB definition (2019).

Region	OWOB	EBP	Comorbidity
National	48.3 (47.6 - 48.9)	12.3 (11.2 - 13.2)	52.4 (50.8 - 53.8)
Beijing	45.7 (41.4 - 50.2)	14.4 (6.9 - 23.6)	48.2 (39.4 - 57.8)
Tianjin	54.6 (51.1 - 59.0)	26.5 (20.8 - 32.9)	62.9 (55.6 - 69.6)
Hebei	41.4 (36.4 - 47.4)	11.6 (5.5 - 19.3)	44.2 (29.3 - 55.8)
Shanxi	48.7 (44.5 - 52.6)	13.8 (9.3 - 19.9)	47.2 (36.4 - 54.7)
Inner Mongolia	56.4 (51.5 - 60.4)	20.7 (15.7 - 26)	61.2 (52.8 - 67.9)
Liaoning	56.6 (50.1 - 61.3)	11.4 (8.1 - 14.5)	58.0 (52.5 - 64.2)
Jilin	53.8 (48.2 - 60.4)	4.5 (-7.8 - 16.4)	50.9 (37.5 - 60.9)
Heilongjiang	59.8 (56.5 - 64.6)	14.5 (5.9 - 23.3)	65.4 (52.7 - 76.2)
Shanghai	53.5 (49.1 - 58.5)	5.9 (0.9 - 10.6)	47.7 (38.7 - 55.6)
Jiangsu	36.7 (30.7 - 42.3)	4.7 (-1.7 - 10.9)	35.6 (26.4 - 49.3)
Zhejiang	36.5 (31.0 - 43.7)	8.5 (2.3 - 13.4)	34.1 (23.3 - 46.1)
Anhui	48.8 (45.6 - 52.8)	8.5 (3.6 - 13.1)	55.6 (49.1 - 60.7)
Fujian	49.4 (44.1 - 54.6)	16.5 (8.9 - 24.1)	62.0 (53.7 - 73.1)
Jiangxi	44.3 (40.0 - 50.1)	10.6 (2.6 - 17.6)	54.5 (45.1 - 65.6)
Shandong	49.2 (44.3 - 54.1)	13.8 (7.1 - 21.2)	55.4 (46.1 - 66.3)
Henan	51.6 (46.3 - 57.6)	7.7 (-7.2 - 17.7)	43.9 (31.5 - 56.1)
Hubei	49.0 (44.6 - 56.5)	6.7 (2.7 - 13.2)	49.9 (40.5 - 59.2)
Hunan	37.2 (33.6 - 41.6)	6.1 (-0.6 - 12.7)	46.5 (37.7 - 55.5)
Guangdong	46.0 (40.6 - 49.9)	10.9 (7.3 - 17)	45.3 (37.4 - 53.6)
Guangxi	51.3 (47.1 - 56.1)	12.5 (5.2 - 21.8)	56.5 (45.7 - 74.2)
Hainan	44.1 (37.1 - 50.6)	1.7 (-1.2 - 5.6)	33.0 (25.0 - 40.5)
Chongqing	40.8 (36.4 - 45.5)	4.6 (-2.1 - 16.8)	51.6 (40.1 - 61.4)
Sichuan	45.7 (39.6 - 51.0)	10.8 (4.0 - 17.0)	47.7 (33.7 - 64.6)
Guizhou	51.7 (47.0 - 56.6)	4.7 (-0.9 - 11.6)	50.0 (42 - 58.7)
Yunnan	50.6 (46.2 - 55.5)	6.7 (1.3 - 14.0)	53.0 (38.2 - 62.3)
Shaanxi	60.2 (54.6 - 66.0)	15.1 (4.9 - 31.5)	63.0 (50.9 - 76.7)
Gansu	51.3 (46.7 - 56.4)	9.6 (5.5 - 14.4)	54.5 (47.1 - 66.5)
Qinghai	49.8 (38.5 - 55.9)	6.7 (3.2 - 9.5)	54.5 (47.9 - 59.8)
Ningxia	46.9 (39.7 - 54.1)	-0.7 (-9.4 - 6.6)	41.2 (29.4 - 48.6)
Xinjiang	48.9 (41.9 - 54.0)	3.8 (-4.9 - 15.4)	45.9 (33.5 - 59.5)

This table presents PAF estimates where overweight/obesity was defined according to the US CDC 2000 growth charts. OWOB: overweight/obesity defined by CDC 2000 criteria; EBP: elevated blood pressure; Comorbidity: co-occurrence of OWOB and EBP.
